# The triglyceride-glucose index: a novel predictor of stroke and all-cause mortality in liver transplantation recipients

**DOI:** 10.1186/s12933-023-02113-x

**Published:** 2024-01-13

**Authors:** Zhendong Ding, Mian Ge, Yuexiang Tan, Chaojin Chen, Ziqing Hei

**Affiliations:** 1 Department of Anesthesiology, The Third Affiliated Hospital of Sun Yat-sen University, No. 600 Tianhe Road, Guangzhou, 510630 China; 2 SageRAN Technology, No. 9-11 Keyun Road, Guangzhou, 510000 China; 3 Center of Big Data and Artificial Intelligence, The Third Affiliated Hospital of Sun Yat-sen University, No.600 Tianhe Road, Guangzhou, 510630 China

**Keywords:** Liver transplantation, Triglyceride-glucose index, End-stage liver diseases, Perioperative stroke, All-cause mortality.

## Abstract

**Background:**

The triglyceride-glucose (TyG) index, identified as a reliable indicator of insulin resistance (IR), was reported to be associated with stroke recurrence and morbidity in the general population and critically ill patients. However, the relationship in liver transplantation (LT) recipients remains unknown. This study aimed to investigate the correlation between the TyG index and post-LT stroke along with all-cause mortality and further assess the influence of IR on the LT recipients’ prognosis.

**Methods:**

The retrospective cohort study enrolled 959 patients who underwent LT at a university-based medical centre between January 2015 and January 2021. The participants were divided into three groups according to their TyG index tertiles. The primary outcome was post-LT stroke. Multivariate logistic regression, COX proportional hazards regression, and restricted cubic spline RCS were used to examine the association between the TyG index and outcomes in LT recipients.

**Results:**

With a median TyG index of 8.23 (7.78–8.72), 780 (87.18% males) patients were eventually included. The incidence of post-LT stroke was 5.38%, and the in-hospital, 1-year, and 3-year mortality rates were 5.54%, 13.21%, and 15.77%, respectively. Multivariate regression analysis showed an independent association between the TyG index and an increased risk of post-LT stroke [adjusted odds ratio (aOR), 3.398 (95% confidence interval [CI]: 1.371–8.426) *P* = 0. 008], in-hospital mortality [adjusted hazard ratio (aHR), 2.326 (95% CI: 1.089–4.931) *P* = 0.025], 1-year mortality [aHR, 1.668 (95% CI: 1.024–2.717) *P* = 0.039], and 3-year mortality [aHR, 1.837 (95% CI: 1.445–2.950) *P* = 0.012]. Additional RCS analysis also suggested a linear increase in the risk of postoperative stroke with elevated TyG index (*P* for nonlinearity = 0.480).

**Conclusions:**

The TyG index may be a valuable and reliable indicator for assessing stroke risk and all-cause mortality in patients undergoing LT, suggesting its potential relevance in improving risk stratification during the peri-LT period.

**Supplementary Information:**

The online version contains supplementary material available at 10.1186/s12933-023-02113-x.

## Background

Liver transplantation (LT) has become the standard treatment choice for patients with end-stage liver disease (ESLD) [1]. This shift in treatment modalities emphasises the need for an enhanced focus on the long-term management of LT patients, with cerebrovascular disease emerging as a critical concern [2, 3]. Perioperative stroke (PS) is defined as an ischaemic or haemorrhagic cerebral event that occurs during or up to 30 days after surgery and is considered a major cerebrovascular complication in patients undergoing LT [4]. The most common causes of PS are cardiac surgery, neurosurgery, thoracic vascular surgery and transplantation [5]. While PS after LT is relatively rare compared to other perioperative complications [6, 7], patients with PS tend to have worse outcomes than non-stroke patients [8]. In particular, the mortality rate of PS patients is approximately eight times higher than that of comparable non-surgical populations, with a mortality rate of 26% [9], and tends to be worse in LT recipients. Consequently, PS places additional burdens on patients, their caregivers, and the healthcare system [5]. Although some factors such as smoking history, kidney insufficiency, advanced age and hypertension have been suggested to be associated with PS [9], the accuracy of these factors in predicting PS incidence remains uncertain. Moreover, there is a paucity of data that specifically examine the risk factors for PS among patients undergoing LT.

As a surrogate marker of insulin resistance (IR), the triglyceride-glucose (TyG) index is derived from the fasting blood glucose (FBG) and triglycerides (TG) [10] and provides a means for accessing the status of both lipids with glucose. The TyG index not only proves to be cost-effective and reproducible but also demonstrates better predictive value than FBG or TG alone [11]. Growing evidence indicates a close association between the TyG index and an elevated risk of adverse cardiovascular events in both the general population [12, 13] and high-risk patient cohorts, such as those with hypertension [14] and critically ill patients [15]. IR is characterised by reduced insulin sensitivity in peripheral tissues and contributes to many metabolic abnormalities associated with critical illnesses [16]. IR also plays an important role in the pathophysiology of microangiopathy, macroangiopathy, neuropathy, and organ failure in critically ill patients [17]. Previous evidence have revealed that critically ill patients experienced severe IR after intensive care unit (ICU) admission, which correlated with their severity rather than their diagnoses [18]. Patients undergoing LT often experience ESLD [19], leading to severe disturbances in lipid metabolism and glucose status [20], with prolonged perioperative intensive care in ICU. Published studies have suggested that TG, diabetes, and IR could be potential risk factors for PS [21, 22]. Consequently, recognising and addressing perioperative IR in LT recipients is crucial for preventing and managing postoperative cardiovascular complications.

Numerous studies have shown that the TyG index is capable of predicting the recurrence and morbidity of strokes [11, 23] and that all-cause mortality tends to increase significantly when the TyG index is elevated [24]. However, it remains unclear whether this association persists in LT recipients. Therefore, this study aimed to explore whether the TyG index could be a potential predictor in LT recipients and assist in identifying individuals at high risk of PS and all-cause mortality for healthcare management and perioperative decision-making.

## Methods

### Study design, setting and population

**Fig. 1:**
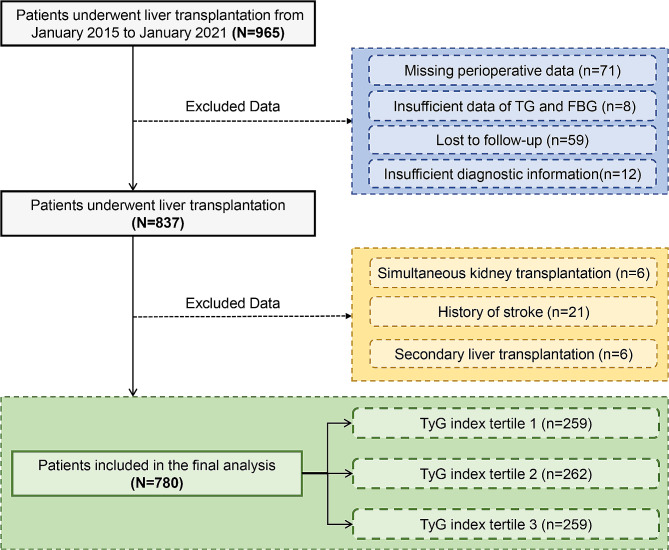
Patient inclusion and exclusion criteria flowchart. FBG fasting blood glucose, TyG triglyceride-glucose

The retrospective cohort study examined the perioperative data of patients who underwent LT retrieved from a perioperative database platform and an electronic health record system at a university-affiliated medical centre between January 2015 and January 2021. Inclusion criteria included: (1) age > 18 years and (2) allogeneic LT. Exclusion criteria encompassed: (1) missing perioperative data, (2) insufficient TG and FBG data before LT, (3) loss to follow-up, (4) insufficient diagnostic information on stroke, (5) a history of stroke, (6) simultaneous kidney and liver transplantations, and (7) secondary LT. Ultimately, 780 patients were enrolled in this study and divided into three groups according to the tertiles of the TyG index (Fig. 1).

This study was approved by the Ethics Committee of the Third Affiliated Hospital of Sun Yat-sen University [27 July 2022, No. (2019)02-609-04]) and was granted exemption from the need for informed consent owing to its retrospective design. This study adhered to the guidelines outlined in the STROBE statement and the Declaration of Helsinki. All the LT recipients included in the study were formally registered in the China Organ Transplant Response System.

### Data collection

Potential variables were divided into five categories: (1) demographics, such as age, sex, American Society of Anaesthesiologists (ASA) classification, body mass index (BMI), smoking, alcoholism, and history of previous surgery; (2) comorbidities, including hypertension, diabetes, respiratory diseases, pulmonary hypertension, renal insufficiency, hypersplenism, cirrhosis, hepatitis B, hepatitis C, and liver cancer; (3) treatments, including haemodialysis, plasma exchange (PE), and mechanical ventilation (MV); (4) laboratory indicators, including white blood cell (WBC), platelets, red blood cell (RBC), aspartate aminotransferase (AST), alanine aminotransferase (ALT), albumin, blood urea nitrogen (BUN), serum creatinine (SCr), international normalised ratio (INR), prothrombin time (PT), FBG, TG, amylase, ammonia, etc.; (5) admission severity of illness scores, including the Model for End-Stage Liver Disease Score (MELD), Child-Pugh score, and the Sepsis-related Organ Failure Assessment Score (SOFA); (6) intraoperative indicators, including emergency surgery, day or night surgery, surgery technique, surgery duration, anhepatic phase duration, massive transfusion, massive blood loss, urinary oliguria, and cardiac arrest. Notably, the TG index was calculated using the formula ln (fasting TG (mg/dl) × FBG (mg/dl])/2 [25].

### Clinical outcomes and follow-up

The primary outcome was the first occurrence of postoperative stroke. The secondary outcomes were the individual endpoints of all-cause mortality, defined as death from any cause during the postoperative hospitalisation period and at one year and three years after LT.

The diagnosis of stroke was confirmed through a comprehensive medical review of imaging reports, and each patient underwent evaluation based on World Health Organization criteria by a certified neurologist without prior access to their medical records [21, 26]. Follow-up visits were scheduled for one year and three years post-LT. At each follow-up visit, details on postoperative anti-rejection therapy and survival status were recorded. In instances where direct contact with the patient was unattainable, telephone interviews were conducted with relatives or caregivers. However, if the patients’ relatives could not be contacted, the patients were considered lost to follow-up and documented in the electronic tracking system.

### Statistical analysis

The normality of continuous parameters was assessed by Kolmogorov-Smirnov test. Depending on the data distributions, continuous variables are presented as mean ± SD or median (interquartile range), while categorical variables are expressed as number (proportion). Continuous variables were analysed using the t-test if they exhibited a normal distribution, while the Kruskal-Wallis test was used for non-normal distributions. The categorical variables were analyzed using chi-square test or Fisher’s exact test. For missing values in the original dataset (Supplementary Table 1, Additional File 1), no categorical variables were identified as missing, and all continuous variables missing at moderate rates (< 10%) were replaced by multiple imputations using the R software. The Kaplan-Meier method was employed to assess the incidence of survival endpoints, and differences based on the TyG index were determined using the log-rank test.

Binary logistic regression was performed to evaluate risk factors and calculate odds ratios (OR) and 95% confidence intervals (CI) between the TyG index and post-LT stroke. Cox proportional hazard models were then used to estimate the hazard ratio (HR) and 95% CI between the TyG index and all-cause death after LT. Confounders included baseline characteristics selected by *P*-value < 0.05 in the univariate analysis and *P*-value < 0.01 in the additional binary logistic regression. Additionally, variables associated with aetiology and prognosis based on recent studies were also included in the multivariate models [21, 27, 28], and the variance inflation factor (VIF) was used as a measure of multicollinearity for all covariates in the multivariate models.

Four models were systematically constructed for analysis: an unadjusted model; model 1, adjusted for sex, age, BMI, and ASA; model 2, adjusted for sex, age, BMI, ASA, hypertension, diabetes, renal insufficiency, HE, MELD score, haemodialysis, HB, WBC, and platelet count; and model 3, adjusted for sex, age, BMI, ASA, diabetes, hypertension, renal insufficiency, HE, MELD score, haemodialysis, HB, WBC, platelet count, day-or-night surgery, surgery duration, intraoperative massive transfusion, massive blood loss, urinary oliguria, and cardiac arrest. Additionally, a restricted cubic spline (RCS) regression model with four knots (5th, 35th, 65th, and 95th) was used to flexibly model possible nonlinear associations between the TyG index and stroke.

The TyG index was categorised into three groups (the first group was used as the reference group) and modelled as a continuous variable in the analyses. *P*-values for the trends were calculated based on the TyG index for each group. To determine the effectiveness of the TyG index as a prognostic indicator, stratified analyses were performed based on sex, hypertension, diabetes, renal insufficiency, hypersplenism, previous surgery, and day or night surgery. Likelihood ratio tests were conducted to examine interactions between the TyG index and the variables utilized for stratification.

Several sensitivity analyses were conducted to validate the robustness of the current results. First, to explore the potential impact of imputation strategies, we repeated the missing value processing using a dataset without imputation and another dataset where imputation involved using the median (for numeric characteristics) or mode (for categorical characteristics). Second, to remove bias from excessively outdated medical records, we excluded patients who underwent LT before 2016. Third, we excluded patients with a history of smoking or alcohol consumption at baseline. Fourth, patients with a preoperative diagnosis of hepatic encephalopathy were excluded. Finally, to avoid the potential influence of surgery-related confounders on the outcomes, we excluded patients who did not undergo piggyback LT.

A two-sided *P*-value < 0.05 was defined as statistical significance. All analyses were performed using the R software (version 4.2.0).

## Results

The enrollment flowchart is presented in Fig. 1. A total of 780 patients who underwent LT were eventually included in this study. The median age of the included patients was 49 (42–56) years, and 680 (87.18%) were male. The median TyG index for all included patients was 8.23 (7.78–8.72). The incidence of stroke after LT was 5.38%, and the in-hospital, 1-year, and 3-year mortality rates were 5.54%, 13.21%, and 15.77%, respectively (Table 1).

**Table 1:** Key baseline clinical characteristics of patients with stratification by stroke

	All (N = 780)	Non-Stroke (N = 738)	Stroke (N = 42)	*P-value*
Age (years)	49.00 (42.00–56.00)	49.00 (41.25–55.75)	52.00 (42.50–61.00)	0.094
Sex (male)	680 (87.18%)	643 (87.1%)	37 (88.1%)	0.855
BMI	22.70 (21.00-24.42)	22.70 (20.92–24.37)	22.50 (21.22–24.78)	0.856
ASA				**< 0.001**
II	71 (9.10%)	70 (9.5%)	1 (2.4%)	
III	706 (90.51%)	668 (90.5%)	38 (90.5%)	
IV	3 (0.38%)	0 (0.0%)	3 (7.1%)	
Smoking	243 (31.15%)	225 (30.5%)	18 (42.9%)	0.092
Alcoholism	200 (25.64%)	184 (24.9%)	16 (38.1%)	0.057
Drug abuse	4 (0.51%)	4 (0.5%)	0 (0.0%)	0.632
Previous surgery	53 (6.79%)	49 (6.6%)	4 (9.5%)	0.47
Child Pugh score	10.00 (8.00–11.00)	10.00 (8.00–11.00)	10.00 (9.00–11.00)	**0.035**
SOFA	11.00 (9.00–13.00)	11.00 (9.00–13.00)	12.00 (10.25-14.00)	**0.042**
MELD	23.00 (22.00–34.00)	22.00 (22.00–34.00)	31.50 (23.25–39.50)	**0.001**
Comorbidities				
Alcoholic liver disease	55 (7.05%)	48 (6.5%)	7 (16.7%)	**0.012**
Cirrhosis	642 (82.31%)	617 (83.6%)	25 (59.5%)	**< 0.001**
Portal hypertension	426 (54.62%)	411 (55.7%)	15 (35.7%)	**0.011**
Hypersplenism	416 (53.33%)	404 (54.7%)	12 (28.6%)	**< 0.001**
Fever	95 (12.18%)	85 (11.5%)	10 (23.8%)	**0.018**
Renal insufficiency	207 (26.54%)	191 (25.9%)	16 (38.1%)	**0.081**
Diabetes	109 (13.97%)	98 (13.3%)	11 (26.2%)	**0.019**
Hypertension	67 (8.59%)	63 (8.5%)	4 (9.5%)	0.824
HE	156 (20.00%)	140 (19.0%)	16 (38.1%)	**0.003**
**Treatments**				
Mechanical ventilation	60 (7.69%)	53 (7.2%)	7 (16.7%)	**0.025**
Hemodialysis	230 (29.49%)	205 (27.8%)	25 (59.5%)	**< 0.001**
PE	170 (21.79%)	151 (20.5%)	19 (45.2%)	**< 0.001**
**Laboratory tests**				
TYG index	8.23 (7.78–8.72)	8.21 (7.76–8.69)	8.68 (8.14–9.02)	**< 0.001**
Hemoglobin (g/L)	101.00 (83.00-122.00)	102.00 (83.00-122.57)	90.14 (75.25-106.25)	**0.003**
WBC (10^9^/L)	5.39 (3.58–8.76)	5.29 (3.55–8.55)	8.18 (3.96–11.82)	**0.015**
Platelet (10^9^/L)	72.52 (46.00-122.00)	73.00 (47.00-123.75)	58.50 (40.00–99.00)	0.063
FBG (mmol/L)	5.00 (4.22–6.43)	4.96 (4.20–6.27)	6.81 (4.75–10.04)	**< 0.001**
TC (mmol/L)	3.03 (2.08–3.93)	3.06 (2.10-4.00)	2.46 (1.90–3.41)	**0.01**
HDL (mmol/L)	0.47 (0.15–0.91)	0.48 (0.15–0.93)	0.34 (0.13–0.60)	**0.02**
FIB (g/L)	1.59 (1.05–2.65)	1.62 (1.07–2.67)	1.27 (0.82–1.89)	**0.004**
ALT (U/L)	54.90 (27.00-116.00)	54.00 (27.00-116.00)	83.85 (29.25-119.75)	**0.005**
IBIL (µmol/L)	38.83 (10.80-137.99)	36.40 (10.50-132.62)	111.60 (25.02–188.40)	**0.026**
SCr (µmol/L)	73.00 (60.00–92.00)	73.00 (60.00–91.00)	73.50 (59.50-148.75)	**0.013**
**Intraoperative indicators**				
Night surgery	276 (35.38%)	251 (34.0%)	25 (59.5%)	**< 0.001**
Surgery duration	521.00 (470.00-580.00)	520.00 (470.00-580.00)	532.50 (486.00-622.25)	0.174
Donor type				**0.005**
DBD	471 (60.38%)	453 (61.4%)	18 (42.9%)	
DCD	301 (38.59%)	279 (37.8%)	22 (52.4%)	
DBCD	8 (1.03%)	6 (0.8%)	2 (4.8%)	
Massive transfusion	231 (29.62%)	210 (28.5%)	21 (50.0%)	**0.003**
Massive blood losing	37 (4.74%)	30 (4.1%)	7 (16.7%)	**< 0.001**
Urinary oliguria	32 (4.10%)	25 (3.4%)	7 (16.7%)	**< 0.001**
Cardiac arrest	16 (2.036%)	13 (1.762%)	3 (7.143%)	**0.017**

*Note*: Data were expressed as mean (standard deviation), median (interquartile range) or n (%). Bold data indicates significance at < 0.05

*Abbreviation*: BMI, body mass index; ASA, American Society of Anesthesiologists; SOFA, sequential organ failure assessment score; MELD, model for end-stage liver disease score; HE, hepatic encephalopathy; PE, plasma exchange; TyG, triglyceride-glucose index; WBC, white blood cell; TG, triglyceride; FBG, fasting blood glucose; TC, total cholesterol; HDL, high density lipoprotein; LDL, low density lipoprotein; PT, prothrombin time; INR, international normalized ratio; FIB, fibrinogen; ALT, alanine aminotransferase; AST, aspartate aminotransferase; TBIL, total bilirubin; IBIL, indirect bilirubin; SCr, serum creatinine; BUN, blood urea nitrogen; DBD, donation after brain death; DCD, donation after circulatory death; DBCD, donation after brain death followed by circulatory death

## Baseline characteristics

A comparison of the baseline characteristics of patients with and without post-LT stroke is shown in Table 1 and Supplementary Table 2 (Additional File 2). The demographic characteristics did not differ significantly between patients with and without post-LT stroke (*P* > 0.05). However, individuals in the stroke group exhibited higher MELD scores; an increased prevalence of alcoholic liver disease, preoperative fever, renal insufficiency, diabetes, and HE; and higher values of WBC, FBG, ALT, IBIL, and SCr. Moreover, mechanical ventilation (MV), haemodialysis, plasma exchange (PE), night surgery, donation after circulatory death (DCD) grafts, intraoperative massive transfusion, massive blood loss, urinary oliguria, and cardiac arrest were significantly associated with post-LT stroke (*P* < 0.05). Specifically, the TyG index level was significantly elevated in the stroke group compared to the non-stroke group [8.68 (8.14–9.02) vs. 8.21 (7.76–8.69), *P* < 0.001].

The baseline characteristics of LT recipients according to the TyG index tertiles are shown in Table 2. All three groups were classified according to TyG index levels [tertile (T)1: (< 7.92), T2: (7.92–8.53), T3: (> 8.53)], with median TyG index levels of 7.62 (7.34–7.77), 8.23 (8.08–8.37), and 8.96 (8.72–9.34), respectively. Notably, with the increase in TyG index levels, patients tended to be older, had a history of diabetes, had higher severity of MELD scores, higher levels of haemoglobin, WBC, and platelets; and a higher incidence of intraoperative urinary oliguria compared to the lower group (*P* < 0.05). In contrast, the percentage of individuals receiving massive transfusions during surgery exhibited a lower TyG index (*P* < 0.05).

**Table 2:** Baseline clinical characteristics and outcomes of patients categorized by TyG index^a^

	All (N = 780)	T1 (N = 259)	T2 (N = 262)	T3 (N = 259)	*P-value*
Age (years)	49.00 (42.00–56.00)	46.00 (39.00–53.00)	50.00 (43.25-56.00)	50.00 (42.00–57.00)	**< 0.001**
Sex (male)	680 (87.18%)	228 (88.03%)	225 (85.88%)	227 (87.64%)	0.735
BMI	22.70 (21.00-24.42)	22.50 (20.80–24.20)	22.57 (20.83–24.37)	23.00 (21.15–24.70)	0.142
ASA					**0.005**
II	71 (9.10%)	13 (5.02%)	29 (11.07%)	29 (11.20%)	
III	706 (90.51%)	246 (94.98%)	233 (88.93%)	227 (87.64%)	
IV	3 (0.38%)	0 (0.00%)	0 (0.00%)	3 (1.16%)	
Previous surgery	53 (6.79%)	21 (8.11%)	15 (5.73%)	17 (6.56%)	0.549
MELD	23.00 (22.00–34.00)	22.00 (22.00-34.50)	22.00 (22.00–30.00)	29.00 (22.00-36.50)	**< 0.001**
Renal insufficiency	207 (26.54%)	76 (29.34%)	62 (23.66%)	69 (26.64%)	0.340
Diabetes	109 (13.97%)	15 (5.79%)	40 (15.27%)	54 (20.85%)	**< 0.001**
Hypertension	67 (8.59%)	18 (6.95%)	27 (10.31%)	22 (8.49%)	0.392
HE	156 (20.00%)	54 (20.85%)	46 (17.56%)	56 (21.62%)	0.468
Hemodialysis	230 (29.49%)	80 (30.89%)	70 (26.72%)	80 (30.89%)	0.483
TYG index	8.23 (7.78–8.72)	7.62 (7.34–7.77)	8.23 (8.08–8.37)	8.96 (8.72–9.34)	**< 0.001**
Hemoglobin (g/L)	101.00 (83.00-122.00)	91.00 (78.00-108.00)	108.00 (87.00-124.00)	107.00 (85.00-132.03)	**< 0.001**
WBC (10^9^/L)	5.39 (3.58–8.76)	4.83 (3.04–7.79)	4.96 (3.52–8.19)	6.52 (4.27–10.15)	**< 0.001**
Platelet (10^9^/L)	72.52 (46.00-122.00)	61.00 (39.50–90.00)	74.00 (49.00-126.75)	92.00 (52.00-146.50)	**< 0.001**
Day-or-Night surgery					0.841
Day	504 (64.62%)	171 (66.02%)	168 (64.12%)	165 (63.71%)	
Night	276 (35.38%)	88 (33.98%)	94 (35.88%)	94 (36.29%)	
Surgery duration	521.00 (470.00-580.00)	530.00 (480.00-585.00)	515.00 (465.00-570.00)	520.00 (469.50–585.00)	0.145
Massive transfusion	231 (29.62%)	102 (39.38%)	61 (23.28%)	68 (26.25%)	**< 0.001**
Massive blood losing	37 (4.74%)	18 (6.95%)	7 (2.67%)	12 (4.63%)	0.071
Urinary oliguria	32 (4.10%)	9 (3.47%)	7 (2.67%)	16 (6.18%)	0.108
Cardiac arrest	16 (2.04%)	6 (2.32%)	7 (2.67%)	3 (1.16%)	0.445
**Outcomes**					
Stroke	42 (5.38%)	8 (3.09%)	10 (3.82%)	24 (9.27%)	**< 0.001**
Hospital morality	43 (5.54%)	11 (4.31%)	11 (4.20%)	21 (8.11%)	**0.027**
1-year morality	103 (13.21%)	31 (11.97%)	28 (10.69%)	44 (16.99%)	**0.028**
3-year morality	123 (15.77%)	35 (13.51%)	36 (13.74%)	52 (20.08%)	**0.020**

*Note*: Data were expressed as mean (standard deviation), median (interquartile range) or n (%). Bold data indicates significance at < 0.05

^a^TyG index: T1 (< 7.92), T2 (7.92–8.53), T3 (> 8.53)

*Abbreviation*: BMI, body mass index; ASA, American Society of Anesthesiologists; MELD, model for end-stage liver disease score; HE, hepatic encephalopathy; TyG, triglyceride-glucose index; WBC, white blood cell

### Correlation between the TyG index and primary outcome

Supplementary Table 3 (Additional File 3) shows the results of logistic regression for the risk of stroke among patients who underwent LT. Independent variables for binary logistic regression included variables that showed significance in the univariate analysis (*P* < 0.05), as well as factors derived from clinical experience and previous research that were thought to influence the occurrence of postoperative stroke. The results show that age, ASA classification, MELD scores, diabetes, TYG index, haemoglobin level, WBC, platelets, night surgery, intraoperative massive transfusion, massive blood loss, and cardiac arrest were influential factors (*P* < 0.01).

**Fig. 2:**
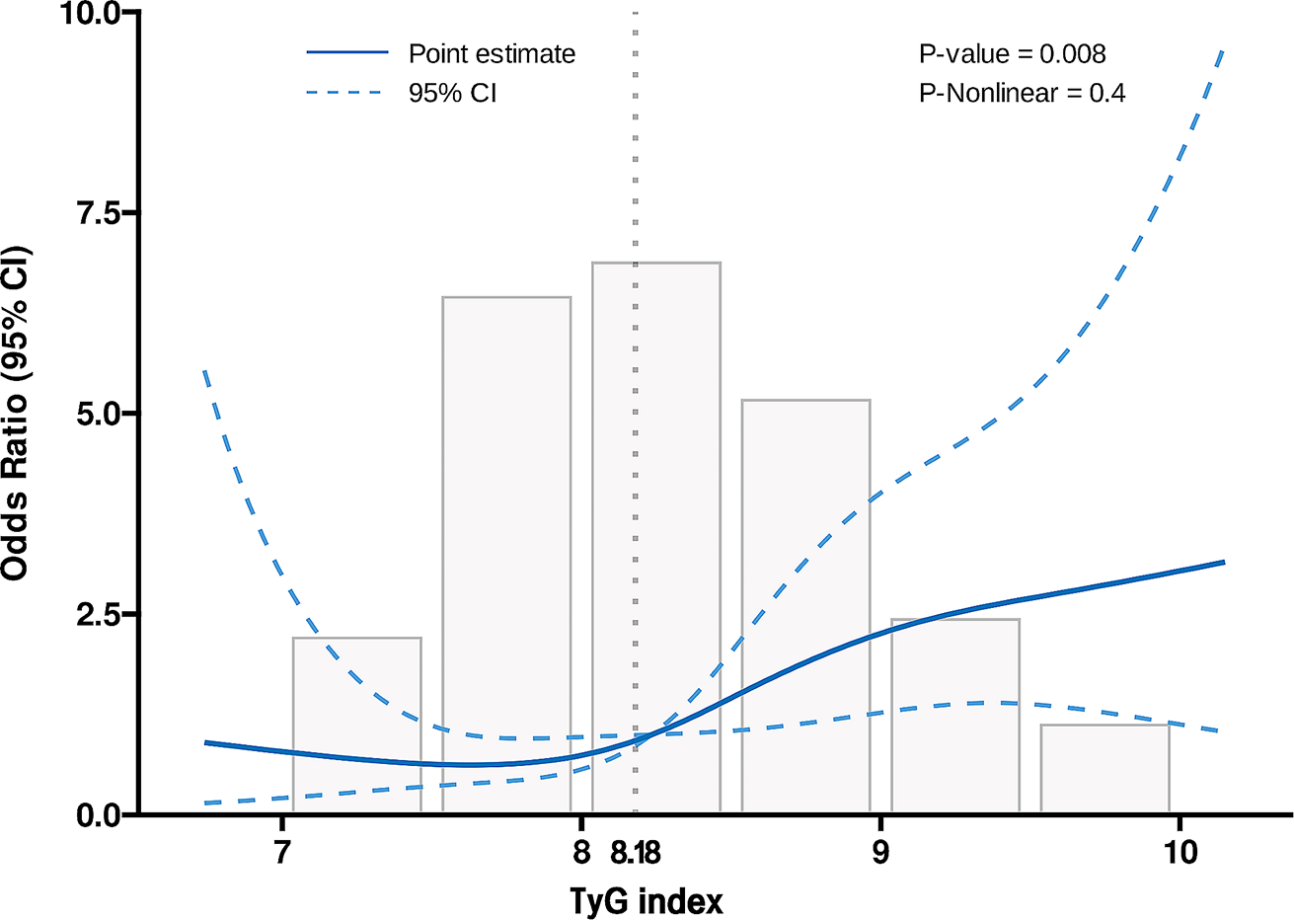
Restricted cubic spline regression analysis of TyG index with postoperative stroke. The heavy central lines in the graph depict the estimated adjusted odds ratios, while the light dotted lines indicate the corresponding 95% confidence intervals. The TyG index 8.18 was chosen as the reference level and is represented by the vertical dotted lines. TyG triglyceride-glucose, CI confidence intervals

An additional logistic regression model was used to investigate the correlation between the TyG index and post-LT stroke. When utilised as a continuous variable, the TyG index level emerged a significant risk factor for post-LT stroke in the unadjusted model [OR, 1.919 (95% CI: 1.280–2.875) *P* = 0.001], partially adjusted model 1 [adjusted OR (aOR), 1.915 (95% CI: 1. 227–2.986) *P* = 0.004], partially adjusted model 2 [aOR, 1.905 (95% CI: 1.183–3.066) *P* = 0.008], and fully adjusted model 3 [aOR, 1.899 (95% CI: 1.180–3.055) *P* = 0.007]. In the context of a nominal variable, it was observed that, compared with patients in the reference group (T1), patients in the high TyG index group (T3) showed a significantly increased risk of post-LT stroke in all four established logistic regression models, as indicated by the following results: unadjusted model [OR, 3. 204 (95% CI: 1.412–7.273), *P* = 0.005], partially adjusted model 1 [aOR, 3.055 (95% CI: 1.330–7.019), *P* = 0.008], partially adjusted model 2 [aOR, 3.348 (95% CI: 1.410–7.952), *P* = 0.006], and fully adjusted model 3 [aOR, 3.398 (95% CI: 1.371–8.426), *P* = 0.008]. There was also a trend of increasing risk with the TyG index (Table 3), as shown by the results of the trend test (*P* for trend = 0.012). The RCS regression model (Fig. 2) illustrated a linearly increasing relationship between the TyG index and the risk of post-LT stroke (*P* for nonlinearity = 0.480). Additionally, the RCS curve identified an inflexion point at TyG = 8.18, which represented a critical point in the relationship between TyG and post-LT stroke.

**Table 3:** Association between quartiles of TyG^a^ index with risk of stroke and all-cause mortality

Outcomes	TyG^a^ index	Event N (%)	Unadjusted			Model 1^b^			Model 2^c^			Model 3^d^	
			OR/HR (95% CI)	*P-value*		OR/HR (95% CI)	*P-value*		OR/HR (95% CI)	*P-value*		OR/HR (95% CI)	*P-value*
Storke	continuous	-	1.919 (1.280–2.875)	0.001		1.915 (1.227–2.986)	0.004		1.905 (1.183–3.066)	0.008		1.899 (1.180–3.055)	0.007
	T1	8 (3.10)	Ref.	-		Ref.	-		Ref.	-		Ref.	-
	T2	10 (3.81)	1.245 (0.483–3.206)	0.649		1.171 (0.449–3.058)	0.747		1.328 (0.494–3.572)	0.574		1.583 (0.577–4.538)	0.375
	T3	24 (9.26)	3.204 (1.412–7.273)	0.005		3.055 (1.330–7.019)	0.008		3.348 (1.410–7.952)	0.006		3.398 (1.371–8.426)	0.008
	*P* for trend		0.003			0.011			0.015			0.012	
Inhospital mortality	continuous	-	1.624 (1.103–2.392)	0.014		1.660 (1.126–2.446)	0.011		1.665 (1.129–2.453)	0.010		1.679 (1.136–2.484)	0.009
	T1	11 (4.31)	Ref.	-		Ref.	-		Ref.	-		Ref.	-
	T2	11 (4.20)	1.088 (0.462–2.561)	0.847		1.143 (0.481–2.719)	0.761		1.137 (0.479–2.695)	0.772		1.075 (0.446–2.589)	0.873
	T3	21 (8.11)	2.232 (1.057–4.714)	0.033		2.244 (1.051–4.792)	0.036		2.309 (1.086–4.911)	0.029		2.326 (1.089–4.931)	0.025
	*P* for trend		0.025			0.038			0.030			0.018	
1-year mortality	continuous	-	1.318 (1.019–1.706)	0.035		1.325 (1.026–1.709)	0.031		1.348 (1.023–1.775)	0.033		1.349 (1.021–1.784)	0.035
	T1	31 (11.97)	Ref.	-		Ref.	-		Ref.	-		Ref.	-
	T2	28 (10.69)	0.941 (0.561–1.583)	0.821		0.986 (0.582–1.673)	0.735		1.053 (0.616–1.801)	0.851		1.021 (0.595–1.756)	0.757
	T3	44 (16.99)	1.631 (1.025–2.595)	0.039		1.646 (1.029–2.633)	0.037		1.664 (1.022–2.711)	0.040		1.668 (1.024–2.717)	0.039
	*P* for trend		0.028			0.026			0.024			0.029	
3-year mortality	continuous	-	1.464 (1.143–1.874)	0.002		1.428 (1.103–1.849)	0.007		1.419 (1.078–1.868)	0.013		1.415 (1.074–1.866)	0.014
	T1	35 (13.51)	Ref.	-		Ref.	-		Ref.	-		Ref.	-
	T2	36 (13.74)	1.101 (0.684–1.772)	0.693		1.066 (0.657–1.727)	0.796		1.138 (0.693–1.869)	0.609		1.295 (0.783–2.143)	0.314
	T3	52 (20.08)	1.784 (1.154–2.758)	0.009		1.654 (1.058–2.586)	0.021		1.721 (1.075–2.755)	0.020		1.837 (1.445–2.950)	0.012
	*P* for trend		0.006			0.015			0.013			0.016	

^a^TyG index: T1 (< 7.92), T2 (7.92–8.53), T3 (> 8.53)

^b^Model 1 was adjusted for age, sex, BMI and ASA classification

^c^Model 2 was adjusted for age, sex, BMI, ASA classification, hypertension, diabetes, renal insufficiency, HE, MELD score, hemodialysis, HB, WBC and platelet

^d^Model 3 was adjusted for age, sex, BMI, ASA classification, hypertension, diabetes, renal insufficiency, HE, MELD score, hemodialysis, HB, WBC, platelet, day-or-night surgery, surgery duration, massive transfusion, massive blood losing, urinary oliguria and intraoperative cardiac arrest

The VIF of the confounding factors in the multivariate logistic regression models were all < 2 according to collinearity diagnostics, indicating no multicollinearity among the covariates in the models (Supplementary Table 4, Additional File 4), and all confounders were defined or interpreted in Supplementary Table 5> (Additional File 5).

### Correlation between the TyG index and secondary outcomes

The Cox proportional hazards regression was employed for evaluating the association between the TyG index level and all-cause mortality following LT. In comparison to patients in T1, those in the T3 group exhibited a higher risk of in-hospital death [adjusted HR (aHR), 2.326 (95% CI: 1.089–4.931), *P* = 0.025], 1-year follow-up [aHR, 1.668 (95% CI: 1.024–2.717), *P* = 0.039], and 3-year follow-up [aHR, 1.837 (95% CI: 1.445–2.950), *P* = 0.012] after fully adjusting for potential confounders (Table 3). The results of the trend test (Table 3) also showed a similar tendency, indicating that the risk of hospital mortality, 1-year mortality, and 3-year mortality exhibited an upward trajectory according to the tertiles of the TyG index (*P* for trend = 0.018, 0.029, and 0.016, respectively).

**Fig. 3:**
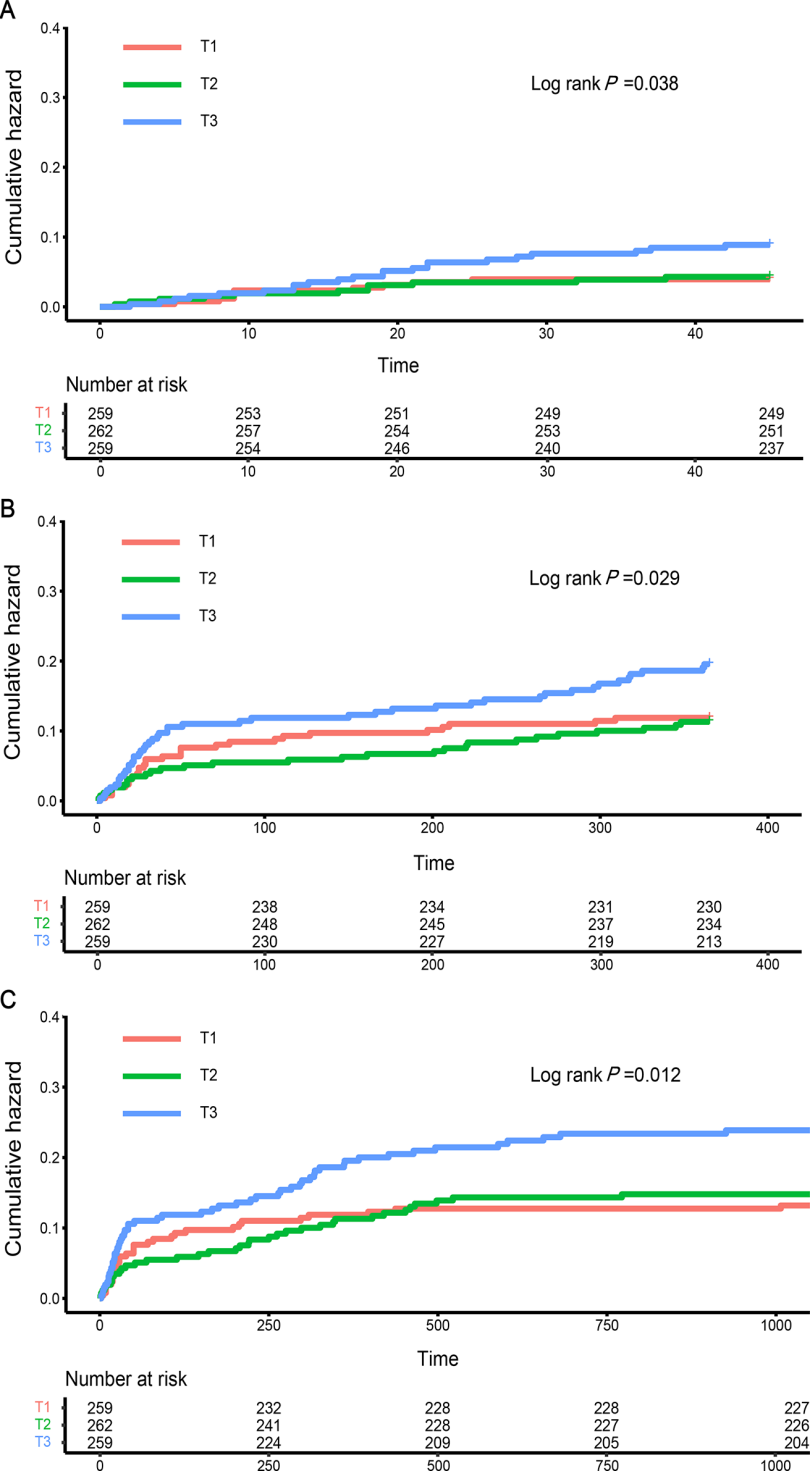
Kaplan–Meier event curves for all-cause death. Kaplan-Meier curves representing the cumulative probability of all-cause death according to groups in hospital (**A**), 1 year (**B**), and 3 years (**C**). Footnote TyG index quartiles: T1 (< 7.92), T2 (7.92–8.53), T3 (> 8.53)

Kaplan-Meier survival analysis (Fig. 3) was utilized to assess the occurrence of all-cause mortality among T1-T3 groups categorized by the TyG index levels. It was observed that patients with a higher TyG index had a significantly increased incidence of all-cause mortality during hospitalisation as well as at the 1-year and 3-year follow-up periods (Log-rank *P* = 0.038, 0.029, and 0.012, respectively).

### Subgroup and sensitivity analysis

**Fig. 4:**
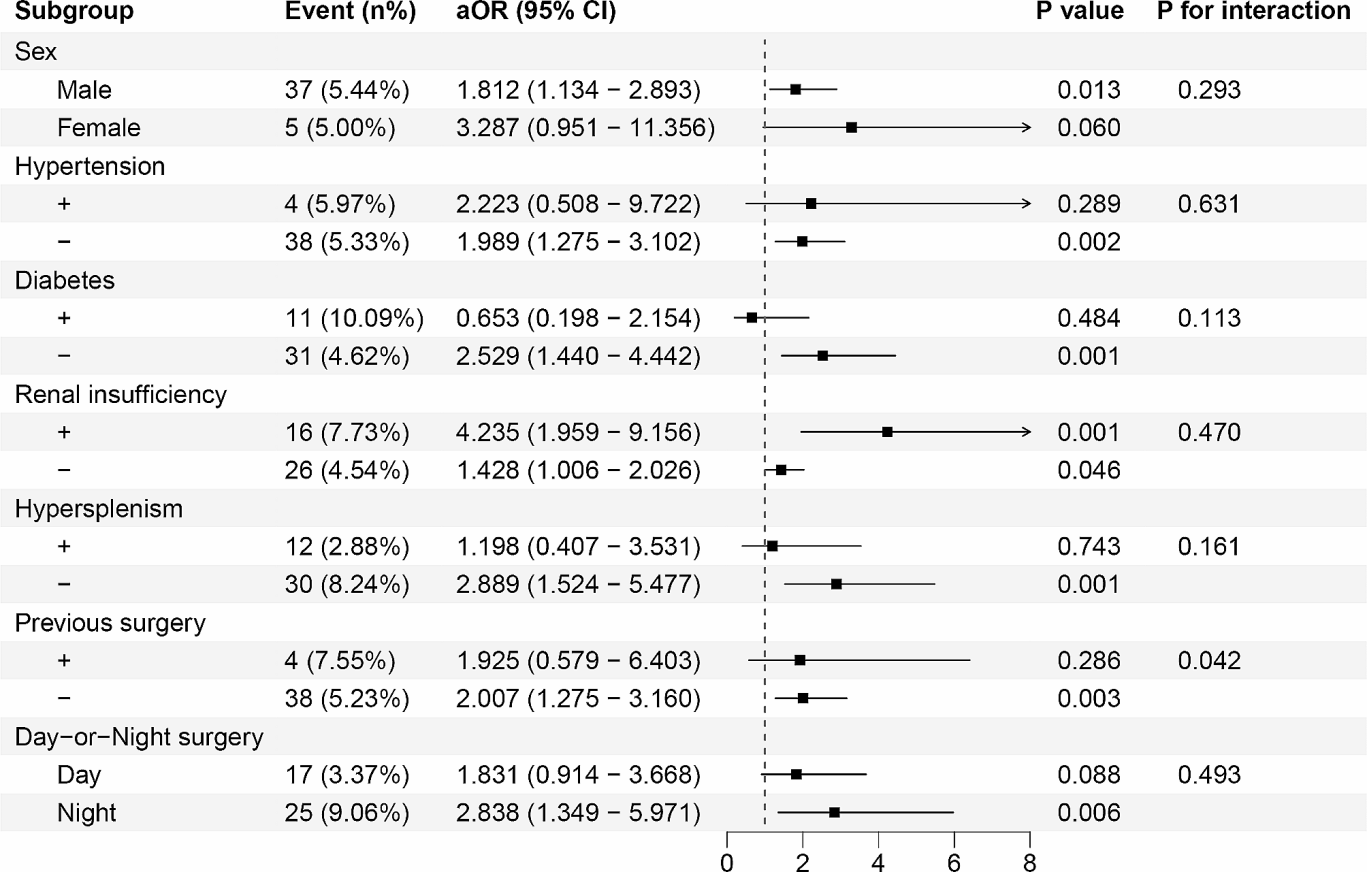
Forest plots of fully adjusted odds ratio for the postopeartive stroke in different subgroups. OR, odds ratio; CI, confidence interval

The TyG index risk stratification value for the primary outcome was further analysed in several subgroups, including sex, hypertension, diabetes, renal insufficiency, hypersplenism, previous surgery, and surgery duration (Fig. 4). The TyG index exhibited a significant association with a higher risk of postoperative stroke in the subgroups of male [aOR, 1.812 (95% CI: (1.134–2.893)], those without hypertension [aOR, 1.989 (95% CI: (1.275–3.102)], those without diabetes [aOR, 2.529 (95% CI: (1.440–4.442)], those with renal insufficiency [aOR, 4.235 (95% CI: (1.959–9.156)], those without hypersplenism [aOR, 2.889 (95% CI: (1.524–5.477)], those without previous surgery [aOR, 2.007 (95% CI: (1.275–3.160)] and those who underwent LT at night [aOR, 2.838 (95% CI: (1.349–5.971)] (all *P* < 0.05). Interestingly, the TyG index appears to exhibit a more pronounced predicting value [aOR (95% CI)] among patients without previous surgery [2.007 (1.275–3.160) vs. 1.925 (0.579–6.403), *P* for interaction = 0.042].

Furthermore, a series of sensitivity analyses were carried out to evaluate the robustness and reliability of our primary results, as shown in Supplementary Table 6 (Additional File 6). First, the results remained robust when using both non-imputed and imputed original datasets, and the TyG index level remained significantly associated with the risk of post-LT stroke in the fully adjusted models (aOR = 2.026, 2.005, respectively). Second, after excluding patients who underwent LT before 2016, our study demonstrated a significant association between the TyG index and the occurrence of post-LT stroke, as evidenced by the unadjusted and fully adjusted models (aOR = 2.002 and 1.955, respectively). Moreover, the results of the analysis, after excluding those patients with smoking history (aOR = 2.427), alcohol consumption (aOR = 2.137), hepatic encephalopathy (aOR = 2.581), and those who did not receive piggyback liver transplantation (aOR = 1.924), were consistent with the main results.

### Other postoperative relevant outcomes

As the T3 group demonstrated a greater association with adverse clinical outcomes, we compared the differences in several relevant post-LT outcomes and complications between T3 and T1–T2 (Supplementary Table 7, Additional File 7). Compared to the patients in T1–T2 groups, those in the T3 group had a higher demand for haemodialysis treatment (22.57% vs. 43.22%, *P* < 0.001), higher hospitalisation costs (294967.9 vs. 324448.1, *P* = 0.001), and longer postoperative ICU stay (3.40 vs. 2.80, *P* = 0.013). Moreover, patients with higher TyG index were associated with more postoperative complications, including a higher incidence of AKI (61.67% vs. 50.52%, *P* = 0.005) and hepatorenal syndrome (6.56% vs. 1.92%, *P* = 0.005).

## Discussion

In this study, we evaluated the association between the TyG index and PS as well as the all-cause mortality in patients who underwent LT. The main findings were as follows: (1) LT recipients presented with a significantly high incidence of PS (up to 5.38%), and an increased TyG index level was associated with a higher risk of post-LT stroke. This finding remained robust even after adjusting for confounding variables. (2) The TyG index was similarly associated with all-cause mortality in LT recipients. (3) Further investigation of the TyG index and perioperative complications of LT indicated that it may be closely associated with the development and progression of perioperative renal insufficiency. Taken together, our results extend the application of the TyG index to LT-related cerebrovascular complications and demonstrate its potential importance as a simple risk predictor to improve risk stratification and perioperative decision-making.

Previous studies have reported the incidence of PS in LT recipients ranging from 2 to 5% [8, 29, 30], which is generally consistent with the findings of our study. In recent years, the TyG index has gained recognition beyond its initial application in diabetes, showing value in various diseases such as metabolic disorders, cardiovascular diseases, atherosclerotic diseases and even COVID-19 [31–33]. In our study, we identified the TyG index as a novel independent risk factor for post-LT stroke after adjusting for confounders. This finding is in line with previous epidemiological studies showing that the TyG index is predictive of stroke in individuals without diabetes mellitus [34]. In a cohort study, Wang et al. [35] demonstrated a 1.45-fold increased risk of ischaemic stroke among patients in the upper quartile of the TyG index. Similarly, another meta-analysis of over 5 million participants suggested that the TyG index was independently associated with a 1.3-fold increased risk of stroke [36]. Moreover, there is a robust association between the TyG index and poor prognosis in critically ill patients, as demonstrated in several cross-sectional and retrospective studies [15, 24, 25]. Subsequent research has revealed that with each unit increase in the TyG index, the likelihood of in-hospital mortality increases by approximately 30–50% [15, 25, 32]. In the current study, we observed that for each unit increase in the TyG index, the risks of in-hospital, 1-year, and 3-year mortality in LT recipients increased by 67.9%, 34.9%, and 41.5%, respectively. These results are generally consistent with those of previous research, indicating a strong association between an elevated TyG index and increased mortality in patients undergoing LT. It is noteworthy that LT recipients exhibit relatively increased in-hospital mortality compared to critically ill patients. This may be attributed to the complicated surgery, prolonged operation time, ischaemia-reperfusion injury, and perioperative infection experienced by LT patients [2, 37]. Hence, regular monitoring of the TyG index has a potential utility in perioperative practice.

In the subgroup analysis, a consistent and independent association between the TyG index and post-LT stroke was observed in male participants. This finding is partially in line with previous research where the correlation between the TyG index and poor outcomes seemed to be more pronounced in male patients than in females [38]. It is interesting to note that none of the interaction tests performed in these studies reached statistical significance, which is consistent with our results. These results suggest that the influence of sex on the relationship between the TyG index and adverse events may not be clinically significant. Additionally, subgroup analysis showed that the TyG index and post-LT stroke were independently correlated in patients without diabetes or hypertension, without a significant interaction (*P* for interaction = 0.113, 0.631, respectively). These results are similar to previous studies and demonstrate that the prognostic-predictive value of the TyG index was independent of hypertension and diabetes mellitus [38, 39]. Moreover, an association between the TyG index and stroke was not observed in patients with hypersplenism and previous surgery, which could be attributed to the fact that hypersplenism and previous surgery are traditionally recognised as unfavourable risk factors associated with PS. Further stratification analysis reduced the sample size, potentially explaining the lack of significant results when patients without renal insufficiency were included. In the sensitivity analysis, the relationship between post-LT stroke and TyG index level was consistent with the core binary logistic regression analysis results after excluding patients with seven different conditions. All these results collectively demonstrate the stability and reliability of the findings of our study.

Furthermore, we investigated the correlation between the TyG index and several perioperative complications which may be associated with LT. Our study found no relationship between the TyG index and postoperative pulmonary complications (PPCs) in LT recipients. Conversely, previous studies have found that high TyG index levels are associated with an increased incidence of chronic lung disease, respiratory symptoms, and reduced lung function, as well as an increased incidence of other infection-related diseases [32, 40]. Possible reasons for this difference include anti-rejection therapy in LT recipients, which may lead to differences in the underlying mechanism of PPCS compared with the non-operated population. Multicentre studies with large sample sizes are needed to investigate the potential association between the TyG index and lung disease. We also identified a potential correlation between a higher TyG index and the occurrence of AKI and hepatorenal syndrome in patients who underwent LT. Patients in the high TyG index group were more likely to receive postoperative haemodialysis treatment. Our study is partially consistent with a previous multicentre cohort study [38] that demonstrated that high TyG index levels correlate with an increased risk of adverse prognosis among patients with end-stage renal disease. This surprising result provides further evidence for the relationship between the TyG index and the pathophysiology and prognosis of various renal diseases.

The biological mechanisms underlying the association between the TyG index and the occurrence and prognosis of stroke remain unclear. One possible pathway is the IR, a widely demonstrated response to critical illness rather than being disease-specific [18]. This close association could be explained by the relationship between ESLD severity and IR status, as assessed using the TyG index. Macrovascular disease, neuropathy, and organ failure are strongly associated with IR [15], which may ultimately lead to further deterioration in critically ill patients, including LT recipients with ESLD. Secondly, it has been widely demonstrated that IR is associated with endothelial dysfunction, oxidative stress, cardiovascular remodelling, coagulation imbalance, and inflammatory response [41–43], all of which are substantial contributors to the deterioration of LT recipients with ESLD. Third, glycometabolic disorders (GD) associated with IR also occur among critically ill patients without prior diabetes [44], and this pathophysiological condition can also be detected in patients with ESLD [44]. GD can lead to tissue acidosis, reactive oxygen species production, and inflammatory cell infiltration, resulting in severe structural tissue damage, which may partly explain the high risk of PS in LT recipients. Finally, individuals suffering from ESLD are typically associated with a variety of metabolic abnormalities, including IR, malnutrition, osteopenia, hypogonadism associated with IGF-I deficiency [45]. Therefore, disturbances in platelet fuction resulting from these reasons may contribute to the pathogenesis of stroke [46, 47]. However, despite these mechanisms explaining our findings to some extent, it is necessary to validate the causal relationship between the TyG index and cerebrovascular events in future prospective studies with larger sample sizes.

The present study confirmed that the TyG index could be used as an effective predictor of post-LT stroke among LT recipients and is independently associated with the risk of all-cause mortality. However, this study has some limitations that must be acknowledged. First, this was a single-centre retrospective analysis based on an observational study design; therefore, definitive causality could not be established. Multivariate-adjusted regression and subgroup analyses were performed to verify the robustness of the main outcomes. Further studies are needed to investigate whether interventions based on the TyG index have a positive impact on preventing stroke after LT and improving clinical prognosis. Second, we were unable to assess dynamic changes in the index during the perioperative period. Previous studies conducted repeated measurements of the TyG index at specified intervals and found that an index reflecting cumulative exposure to TyG outperformed a single measurement in risk prediction [48]. Therefore, the application of the TyG index at baseline, calculated prior to surgery, may be less robust.

## Conclusions

Our study extended the applicability of the TyG index to LT recipients and demonstrated the potential applicability of the TyG index as an indicator for the risk stratification of PS and all-cause mortality among these patients. Consequently, monitoring the TyG index may improve risk stratification and guide perioperative management. However, further investigation is needed to evaluate whether improved management of the TyG index can provide a better clinical prognosis.

### Electronic supplementary material

Below is the link to the electronic supplementary material.

**Supplementary Material 1: Supplementary Table 1**. Overview of missing values in the original data

**Supplementary Material 2: Supplementary Table 2**. All baseline clinical characteristics of patients stratified by stroke

**Supplementary Material 3: Supplementary Table 3**. Binary logistic regression analysis of the factors influencing stroke of the study population

**Supplementary Material 4: Supplementary Table 4**. Collinearity diagnostics by variance expansion factor (VIF)

**Supplementary Material 5: Supplementary Table 5**. The definitions of confounders

**Supplementary Material 6: Supplementary Table 6**. Association between TyG index and postoperative stroke in sensitivity analyses

**Supplementary Material 7: Supplementary Table 7**. Postoperative relative outcomes of patients categorized by TyG index

**Supplementary Material 8: Supplementary Table 8**. Supplementary Online Content

## Data Availability

The original data supporting the results obtained are available from the corresponding author with reasonable ethical research demands.

## Abbreviations

TyG: triglyceride-glucose index
IR: insulin resistance
LT: liver transplantation
RCS: restricted cubic spline
OR: odds ratio
CI: confidence interval
HR: hazard ratio
ESLD: end-stage liver disease
PS: perioperative stroke
FBG: fasting blood glucose
TG: triglycerides
ICU: intensive care unit
BMI: body mass index
ASA: American Society of Anaesthesiologists
PE: plasma exchange
MV: mechanical ventilation
RBC: red blood cell
WBC: white blood cell
ALT: alanine aminotransferase
AST: aspartate aminotransferase
SCr: serum creatinine
BUN: blood urea nitrogen
INR378: international normalised ratio
PT: prothrombin time
MELD: Model for End-Stage Liver Disease Score
SOFA: Sepsis-related Organ Failure Assessment Score
VIF: variance inflation factor
GD: glycometabolic disorders
