# Supplementary material for: The triglyceride-glucose index: a novel predictor of stroke and all-cause mortality in liver transplantation recipients
Source: Cardiovasc Diabetol. 2024 Jan 13;23:27. doi: 10.1186/s12933-023-02113-x (PMC10787491; doi:10.1186/s12933-023-02113-x)
Supplement: Supplementary file 1 — Supplementary Material 1: Supplementary Table 1. Overview of missing values in the original data [file 12933_2023_2113_MOESM1_ESM.docx]

**Supplementary Table 1.** Overview of missing values in the original data.

| **Variables** | **Missing number (%)** | **Variables** | **Missing number (%)** |
| --- | --- | --- | --- |
| Age | 0 | HE | 0 |
| Sex | 0 | Metabolic acidosis | 0 |
| Height | 1.7 | **Treatments** |  |
| Weight | 1.7 | Mechanical ventilation | 0 |
| BMI | 3.3 | Hemodialysis | 0 |
| ASA | 0 | PE | 0 |
| Smoking | 0 | **Laboratory tests** |  |
| Alcoholism | 0 | TYG index | 0 |
| Drug abuse | 0 | Hemoglobin | 7.3 |
| Previous surgery | 0 | WBC | 1.0 |
| Child Pugh score | 7.9 | Platelet | 1.2 |
| SOFA | 8.5 | Amylase | 6.5 |
| MELD | 5.8 | TG | 0 |
| Comorbidities | 0 | FBG | 0 |
| hepatitis B | 0 | TC | 0 |
| hepatitis C | 0 | HDL | 0 |
| Liver cancer | 0 | LDL | 0 |
| Alcoholic liver disease | 0 | PT | 7.4 |
| Cirrhosis | 0 | INR | 1.7 |
| Portal hypertension | 0 | FIB | 1.2 |
| Hypersplenism | 0 | ALT | 8.6 |
| Fever | 0 | AST | 8.7 |
| Renal insufficiency | 0 | TBIL | 7.2 |
| Diabetes | 0 | IBIL | 7.1 |
| Hypertension | 0 | SCr | 0 |
| PAH | 0 | BUN | 7.6 |
| Respiratory disease | 0 | Albumin | 4.1 |
| Ammonia | 5.4 | **Outcomes** |  |
| **Intraoperative indicators** |  | Secondary operation | 0 |
| Emergency surgery | 0 | Hemodialysis | 0 |
| Day-or-Night surgery | 0 | Hospitalization cost (yuan) | 5.4 |
| Surgery duration | 0 | Total length of stay (d) | 0 |
| Blood group incompatibility | 0 | Postoperative length of stay | 0 |
| Donor type | 0 | Postoperative ICU stay (d) | 0 |
| Surgery technique | 0 | PPCs | 0 |
| Anhepatic phase duration | 8.7 | PND | 0 |
| Cold ischemic duration | 8.3 | AKI | 0 |
| Massive transfusion | 0 | Sepsis | 0 |
| Massive blood losing | 0 | Hepatorenal syndrome | 0 |
| Uriry oliguria | 0 |  |  |
| Electrolyte imbalance | 0 |  |  |
| Cardiac arrest | 0 |  |  |
| Hyperlactatemia | 0 |  |  |
| Vasopressor administration | 0 |  |  |

**Abbreviation:** BMI, body mass index; ASA, American Society of Anesthesiologists; SOFA, sequential organ failure assessment score; MELD, model for end-stage liver disease score; HE, hepatic encephalopathy; PE, plasma exchange; TyG, triglyceride-glucose index; WBC, white blood cell; TG, triglyceride; FBG, fasting blood glucose;; TC, total cholesterol; HDL, high density lipoprotein; LDL, low density lipoprotein; PT, prothrombin time; INR, international normalized ratio; FIB, fibrinogen; ALT, alanine aminotransferase; AST, aspartate aminotransferase; TBIL, total bilirubin; IBIL, indirect bilirubin; SCr, serum creatinine; BUN, blood urea nitrogen; PPCs, postoperative pulmonary complications; PND, perioperative neurocognitive dysfunction; AKI, acute kidney insufficiency.
