# Supplementary material for: The triglyceride-glucose index: a novel predictor of stroke and all-cause mortality in liver transplantation recipients
Source: Cardiovasc Diabetol. 2024 Jan 13;23:27. doi: 10.1186/s12933-023-02113-x (PMC10787491; doi:10.1186/s12933-023-02113-x)
Supplement: Supplementary file 2 — Supplementary Material 2: Supplementary Table 2. All baseline clinical characteristics of patients stratified by stroke [file 12933_2023_2113_MOESM2_ESM.docx]

**Supplementary Table 2.** All baseline clinical characteristics of patients stratified by stroke.

|  | **All (N=780)** | **Non-Stroke (N=738)** | **Stroke (N=42)** | ***P****-value* |
| --- | --- | --- | --- | --- |
| Age (years) | 49.00 (42.00-56.00) | 49.00 (41.25-55.75) | 52.00 (42.50-61.00) | 0.094 |
| Sex (male) | 680 (87.18%) | 643 (87.1%) | 37 (88.1%) | 0.855 |
| Height (cm) | 168.00 (165.00-172.00) | 168.00 (165.00-172.00) | 168.00 (165.25-172.00) | 0.894 |
| Weight (kg) | 65.00 (60.00-69.00) | 65.00 (60.00-69.00) | 65.00 (60.00-65.00) | 0.732 |
| BMI | 22.70 (21.00-24.42) | 22.70 (20.92-24.37) | 22.50 (21.22-24.78) | 0.856 |
| ASA |  |  |  | **<0.001** |
| 2 | 71 (9.10%) | 70 (9.5%) | 1 (2.4%) |  |
| 3 | 706 (90.51%) | 668 (90.5%) | 38 (90.5%) |  |
| 4 | 3 (0.38%) | 0 (0.0%) | 3 (7.1%) |  |
| Smoking | 243 (31.15%) | 225 (30.5%) | 18 (42.9%) | 0.092 |
| Alcoholism | 200 (25.64%) | 184 (24.9%) | 16 (38.1%) | 0.057 |
| Drug abuse | 4 (0.51%) | 4 (0.5%) | 0 (0.0%) | 0.632 |
| Previous surgery | 53 (6.79%) | 49 (6.6%) | 4 (9.5%) | 0.47 |
| Child Pugh score | 10.00 (8.00-11.00) | 10.00 (8.00-11.00) | 10.00 (9.00-11.00) | **0.035** |
| SOFA | 11.00 (9.00-13.00) | 11.00 (9.00-13.00) | 12.00 (10.25-14.00) | **0.042** |
| MELD | 23.00 (22.00-34.00) | 22.00 (22.00-34.00) | 31.50 (23.25-39.50) | **0.001** |
| Comorbidities |  |  |  |  |
| hepatitis B | 594 (76.15%) | 567 (76.8%) | 27 (64.3%) | 0.064 |
| hepatitis C | 18 (2.31%) | 18 (2.4%) | 0 (0.0%) | 0.306 |
| Liver cancer | 317 (40.64%) | 306 (41.5%) | 11 (26.2%) | 0.05 |
| Alcoholic liver disease | 55 (7.05%) | 48 (6.5%) | 7 (16.7%) | **0.012** |
| Cirrhosis | 642 (82.31%) | 617 (83.6%) | 25 (59.5%) | **<0.001** |
| Portal hypertension | 426 (54.62%) | 411 (55.7%) | 15 (35.7%) | **0.011** |
| Hypersplenism | 416 (53.33%) | 404 (54.7%) | 12 (28.6%) | **<0.001** |
| Fever | 95 (12.18%) | 85 (11.5%) | 10 (23.8%) | **0.018** |
| Renal insufficiency | 207 (26.54%) | 191 (25.9%) | 16 (38.1%) | **0.081** |
| Diabetes | 109 (13.97%) | 98 (13.3%) | 11 (26.2%) | **0.019** |
| Hypertension | 67 (8.59%) | 63 (8.5%) | 4 (9.5%) | 0.824 |
| Pulmonary arterial hypertension | 3 (0.38%) | 3 (0.4%) | 0 (0.0%) | 0.679 |
| Respiratory disease | 207 (26.54%) | 191 (25.9%) | 16 (38.1%) | 0.081 |
| HE | 156 (20.00%) | 140 (19.0%) | 16 (38.1%) | **0.003** |
| Metabolic acidosis | 313 (40.13%) | 292 (39.6%) | 21 (50.0%) | 0.18 |
| **Treatments** |  |  |  |  |
| Mechanical ventilation | 60 (7.69%) | 53 (7.2%) | 7 (16.7%) | **0.025** |
| Hemodialysis | 230 (29.49%) | 205 (27.8%) | 25 (59.5%) | **<0.001** |
| PE | 170 (21.79%) | 151 (20.5%) | 19 (45.2%) | **<0.001** |
| **Laboratory tests** |  |  |  |  |
| TYG index | 8.23 (7.78-8.72) | 8.21 (7.76-8.69) | 8.68 (8.14-9.02) | **<0.001** |
| Hemoglobin (g/L) | 101.00 (83.00-122.00) | 102.00 (83.00-122.57) | 90.14 (75.25-106.25) | **0.003** |
| WBC (10^9^/L) | 5.39 (3.58-8.76) | 5.29 (3.55-8.55) | 8.18 (3.96-11.82) | **0.015** |
| Platelet (10^9^/L) | 72.52 (46.00-122.00) | 73.00 (47.00-123.75) | 58.50 (40.00-99.00) | 0.063 |
| Amylase (U/L) | 76.00 (54.00-107.00) | 76.00 (55.00-106.00) | 68.00 (49.00-112.50) | 0.482 |
| TG (mmol/L) | 0.74 (0.55-1.05) | 0.74 (0.55-1.05) | 0.77 (0.60-1.04) | 0.677 |
| FBG (mmol/L) | 5.00 (4.22-6.43) | 4.96 (4.20-6.27) | 6.81 (4.75-10.04) | **<0.001** |
| TC (mmol/L) | 3.03 (2.08-3.93) | 3.06 (2.10-4.00) | 2.46 (1.90-3.41) | **0.01** |
| HDL (mmol/L) | 0.47 (0.15-0.91) | 0.48 (0.15-0.93) | 0.34 (0.13-0.60) | **0.02** |
| LDL (mmol/L) | 1.50 (0.96-2.32) | 1.53 (0.96-2.33) | 1.25 (0.94-1.76) | 0.077 |
| PT (s) | 17.75 (14.30-26.40) | 17.60 (14.30-26.40) | 20.15 (15.80-25.80) | 0.068 |
| INR | 1.73 (1.22-2.83) | 1.71 (1.21-2.80) | 2.29 (1.56-3.80) | 0.338 |
| FIB (g/L) | 1.59 (1.05-2.65) | 1.62 (1.07-2.67) | 1.27 (0.82-1.89) | **0.004** |
| ALT (U/L) | 54.90 (27.00-116.00) | 54.00 (27.00-116.00) | 83.85 (29.25-119.75) | **0.005** |
| AST (U/L) | 85.00 (43.00-150.00) | 82.00 (43.00-149.09) | 110.00 (54.25-158.90) | 0.236 |
| TBIL (μmol/L) | 126.55 (24.64-434.47) | 120.14 (24.41-433.00) | 313.52 (67.90-516.54) | 0.137 |
| IBIL (μmol/L) | 38.83 (10.80-137.99) | 36.40 (10.50-132.62) | 111.60 (25.02-188.40) | **0.026** |
| SCr (μmol/L) | 73.00 (60.00-92.00) | 73.00 (60.00-91.00) | 73.50 (59.50-148.75) | **0.013** |
| BUN (mmol/L) | 4.79 (3.51-6.92) | 4.79 (3.51-6.96) | 4.76 (3.40-6.74) | 0.27 |
| Albumin (g/L) | 35.58 (4.91) | 35.63 (4.96) | 34.83 (3.92) | 0.305 |
| Ammonia (μmol/L) | 26.09 (18.61-36.77) | 26.01 (18.58-36.15) | 28.17 (19.65-48.13) | 0.192 |
| **Intraoperative indicators** | |  |  |  |
| Emergency surgery | 161 (20.64%) | 155 (21.0%) | 6 (14.3%) | 0.295 |
| Day-or-Night surgery | 276 (35.38%) | 251 (34.0%) | 25 (59.5%) | **<0.001** |
| Surgery duration | 521.00 (470.00-580.00) | 520.00 (470.00-580.00) | 532.50 (486.00-622.25) | 0.174 |
| Blood group incompatibility | 104 (13.33%) | 95 (12.9%) | 9 (21.4%) | 0.115 |
| Donor type |  |  |  | **0.005** |
| DBD | 471 (60.38%) | 453 (61.4%) | 18 (42.9%) |  |
| DCD | 301 (38.59%) | 279 (37.8%) | 22 (52.4%) |  |
| DBCD | 8 (1.03%) | 6 (0.8%) | 2 (4.8%) |  |
| Surgery technique |  |  |  | 0.147 |
| Piggyback | 718 (92.05%) | 676 (91.6%) | 42 (100.0%) |  |
| Split liver | 33 (4.23%) | 33 (4.5%) | 0 (0.0%) |  |
| Standard | 29 (3.72%) | 29 (3.9%) | 0 (0.0%) |  |
| Anhepatic phase duration | 46.00 (40.00-54.00) | 46.00 (40.00-54.00) | 46.50 (38.50-56.75) | 0.738 |
| Cold ischemic duration | 360.00 (330.00-410.00) | 360.00 (330.00-410.00) | 360.00 (330.00-420.00) | 0.759 |
| Massive transfusion | 231 (29.62%) | 210 (28.5%) | 21 (50.0%) | **0.003** |
| Massive blood losing | 37 (4.74%) | 30 (4.1%) | 7 (16.7%) | **<0.001** |
| Urinary oliguria | 32 (4.10%) | 25 (3.4%) | 7 (16.7%) | **<0.001** |
| Electrolyte imbalance | 302 (38.72%) | 286 (38.8%) | 16 (38.1%) | 0.932 |
| Cardiac arrest | 16 (2.036%) | 13 (1.762%) | 3 (7.143%) | **0.017** |
| Hyperlactatemia | 405 (51.92%) | 388 (52.6%) | 17 (40.5%) | 0.127 |
| Vasopressor administration | 760 (97.44%) | 719 (97.4%) | 41 (97.6%) | 0.938 |

**Note:** Data were expressed as mean (standard deviation), median (interquartile range) or n (%). Bold data indicates significance at <0.05.

**Abbreviation:** BMI, body mass index; ASA, American Society of Anesthesiologists; SOFA, sequential organ failure assessment score; MELD, model for end-stage liver disease score; HE, hepatic encephalopathy; PE, plasma exchange; TyG, triglyceride-glucose index; WBC, white blood cell; TG, triglyceride; FBG, fasting blood glucose;; TC, total cholesterol; HDL, high density lipoprotein; LDL, low density lipoprotein; PT, prothrombin time; INR, international normalized ratio; FIB, fibrinogen; ALT, alanine aminotransferase; AST, aspartate aminotransferase; TBIL, total bilirubin; IBIL, indirect bilirubin; SCr, serum creatinine; BUN, blood urea nitrogen; DBD, donation after brain death; DCD, donation after circulatory death; DBCD, donation after brain death followed by circulatory death.
