# Supplementary material for: The triglyceride-glucose index: a novel predictor of stroke and all-cause mortality in liver transplantation recipients
Source: Cardiovasc Diabetol. 2024 Jan 13;23:27. doi: 10.1186/s12933-023-02113-x (PMC10787491; doi:10.1186/s12933-023-02113-x)
Supplement: Supplementary file 3 — Supplementary Material 3: Supplementary Table 3. Binary logistic regression analysis of the factors influencing stroke of the study population [file 12933_2023_2113_MOESM3_ESM.docx]

**Supplementary Table 3.** Binary logistic regression analysis of the factors influencing stroke of the study population.

| **Variables** | **OR/β** | | **Lower 95% CI** | **Upper 95% CI** | **P-value** |
| --- | --- | --- | --- | --- | --- |
| Age | 1.030 | | 0.999 | 1.061 | 0.055 |
| Sex | 1.093 | | 0.419 | 2.850 | 0.855 |
| BMI | 0.972 | | 0.878 | 1.076 | 0.588 |
| Smoking | 1.710 | | 0.910 | 3.214 | 0.0956 |
| Alcoholism | 1.853 | | 0.972 | 3.531 | 0.061 |
| Previous surgery | 1.481 | | 0.508 | 4.316 | 0.473 |
| Child Pugh score | 1.204 | | 1.025 | 1.415 | 0.023 |
| SOFA | 1.132 | | 1.011 | 1.266 | 0.031 |
| MELD | 1.061 | | 1.022 | 1.101 | **0.002** |
| Alcoholic liver disease | 2.875 | | 1.213 | 6.812 | 0.016 |
| Cirrhosis | 0.288 | | 0.151 | 0.551 | 0.021 |
| Portal hypertension | 0.442 | | 0.231 | 0.844 | 0.013 |
| Hypersplenism | 0.331 | | 0.166 | 0.656 | **0.002** |
| Fever | 2.401 | | 1.139 | 5.058 | 0.021 |
| Renal insufficiency | 1.762 | | 0.925 | 3.356 | 0.084 |
| Diabetes | 2.317 | | 1.128 | 4.761 | 0.022 |
| Hypertension | 1.128 | | 0.389 | 3.262 | 0.824 |
| HE | 2.629 | | 1.373 | 5.032 | **0.004** |
| Mechanical ventilation | 2.585 | | 1.096 | 6.098 | 0.030 |
| Hemodialysis | 3.824 | | 2.023 | 7.228 | **<0.001** |
| PE | 3.211 | | 1.705 | 6.050 | **0.003** |
| TYG index | 2.008 | | 1.311 | 3.073 | **0.001** |
| Hemoglobin | 0.979 | | 0.965 | 0.992 | **0.002** |
| WBC | 1.079 | | 1.029 | 1.131 | **0.002** |
| Platelet | 0.995 | | 0.989 | 1.001 | 0.068 |
| TC | 0.741 | | 0.574 | 0.958 | 0.022 |
| HDL | 0.431 | | 0.192 | 0.970 | 0.041 |
| FIB | 0.649 | | 0.457 | 0.922 | 0.015 |
| ALT | 1.000 | | 0.999 | 1.002 | 0.558 |
| IBIL | 1.003 | | 1.000 | 1.006 | 0.042 |
| SCr | 1.003 | | 1.000 | 1.006 | 0.026 |
| Day-or-Night surgery | | |  |  |  |
| Day | Ref. | | Ref. | Ref. | Ref. |
| Night | 2.853 | | 1.513 | 5.383 | **0.001** |
| Surgery duration | 1.001 | | 0.999 | 1.003 | 0.359 |
| Donor type |  | |  |  |  |
| DBD | Ref. | | Ref. | Ref. | Ref. |
| DCD | 1.984 | | 1.046 | 3.766 | 0.036 |
| DBCD | 8.389 | | 1.582 | 44.484 | 0.012 |
| Massive transfusion | 2.514 | | 1.345 | 4.700 | **0.004** |
| Massive blood losing | 4.720 | | 1.938 | 11.493 | **<0.001** |
| Urinary oliguria | | 5.704 | 2.309 | 14.089 | **<0.001** |
| Cardiac arrest | 4.290 | | 1.174 | 15.680 | 0.027 |

**Abbreviation:** BMI, body mass index; SOFA, sequential organ failure assessment score; MELD, model for end-stage liver disease score; HE, hepatic encephalopathy; PE, plasma exchange; TyG, triglyceride-glucose index; WBC, white blood cell; FBG, fasting blood glucose; HDL, high density lipoprotein; FIB, fibrinogen; ALT, alanine aminotransferase; IBIL, indirect bilirubin; SCr, serum creatinine; DBD, donation after brain death; DCD, donation after circulatory death; DBCD, donation after brain death followed by circulatory death.
