# Supplementary material for: The triglyceride-glucose index: a novel predictor of stroke and all-cause mortality in liver transplantation recipients
Source: Cardiovasc Diabetol. 2024 Jan 13;23:27. doi: 10.1186/s12933-023-02113-x (PMC10787491; doi:10.1186/s12933-023-02113-x)
Supplement: Supplementary file 4 — Supplementary Material 4: Supplementary Table 4. Collinearity diagnostics by variance expansion factor (VIF) [file 12933_2023_2113_MOESM4_ESM.docx]

**Supplementary Table 4.** Collinearity diagnostics by variance expansion factor (VIF).

| variables | VIF |
| --- | --- |
| Age | 1.2 |
| Sex | 1.1 |
| BMI | 1.1 |
| ASA | 1.1 |
| MELD | 1.8 |
| Diabetes | 1.3 |
| Hypertension | 1.3 |
| Renal insufficiency | 1.3 |
| HE | 1.4 |
| Hemodialysis | 1.5 |
| TYG index | 1.9 |
| Hemoglobin | 1.4 |
| WBC | 1.4 |
| Platelet | 1.3 |
| Day-or-Night surgery | 1 |
| Surgery duration | 1.1 |
| Massive transfusion | 1.4 |
| Massive blood losing | 1.2 |
| Urinary oliguria | 1.1 |
| Cardiac arrest | 1.6 |

**Note:** VIF for a variable = 1/(1-R2), where R2 is the R-squared of the regression model of that variable against all other variables (e.g. X1=X2+X3+...). All covariates with VIF ≤ 5.

**Abbreviation:** BMI, body mass index; ASA, American Society of Anesthesiologists; MELD, model for end-stage liver disease score; HE, hepatic encephalopathy; TyG, triglyceride-glucose index; WBC, white blood cell.
