# Supplementary material for: The triglyceride-glucose index: a novel predictor of stroke and all-cause mortality in liver transplantation recipients
Source: Cardiovasc Diabetol. 2024 Jan 13;23:27. doi: 10.1186/s12933-023-02113-x (PMC10787491; doi:10.1186/s12933-023-02113-x)
Supplement: Supplementary file 5 — Supplementary Material 5: Supplementary Table 5. The definitions of confounders [file 12933_2023_2113_MOESM5_ESM.docx]

**Supplementary Table 5.** The definitions of confounders.

| **Confounder** | **Definition** |
| --- | --- |
| **Patient-related confounders** | |
| Age | age in the year of surgery |
| Sex | male or female |
| BMI (kg m^-2^) | calculated by the body weight and height measured at admission |
| ASA classification | assessed by the anaesthesiologists in charge of the case |
| Hypertension | with a previous diagnosis of hypertension, including well-controlled hypertension |
| Diabetes | with a previous diagnosis of diabetes mellitus, including well-controlled diabetes |
| HE | with a previous diagnosis of hepatic encephalopathy, including covert hepatic encephalopathy |
| MELD score | calculated by the SCr, TBIL and INR before surgery |
| Hemodialysis | Renal insufficiency combined with fluid and electrolyte imbalance requiring any form of hemodialysis treatment including continuous renal replacement therapy |
| Hemoglobin | / |
| WBC | / |
| Platelet | / |
| Renal insufficiency | The ratio of preoperative maximum SCr to minimum SCr was over 1.5 in the last test before surgery |
| **Surgery-related confounders** | |
| Day-or-Night surgery | whether the timing of the surgery was during the day (8:00 AM- 8:00 PM) or at night (8:00 PM- 8:00 AM) |
| Surgery duration | duration of the liver transplantation |
| Massive transfusion | transfusion of more than 20 units of red blood cells or transfusion of blood components exceeding 1 to 1.5 times the patient's own blood volume in 24 hours. |
| Massive blood losing | loss of a circulating blood volume in 24 hours, or half the circulating blood volume within 3 hours, or a bleeding rate greater than 150 mL/min. |
| Uriry oliguria | intraoperative urine output ≤0.5ml/kg/h |
| Intra-liver transplantation cardiac arrest | any cardiac arrest that occurs during surgery |

**Abbreviation:** BMI, body mass index; ASA, American Society of Anesthesiologists; HE, hepatic encephalopathy; MELD, model for end-stage liver disease score; WBC, white blood cell.
