# Supplementary material for: The triglyceride-glucose index: a novel predictor of stroke and all-cause mortality in liver transplantation recipients
Source: Cardiovasc Diabetol. 2024 Jan 13;23:27. doi: 10.1186/s12933-023-02113-x (PMC10787491; doi:10.1186/s12933-023-02113-x)
Supplement: Supplementary file 6 — Supplementary Material 6: Supplementary Table 6. Association between TyG index and postoperative stroke in sensitivity analyses [file 12933_2023_2113_MOESM6_ESM.docx]

**Supplementary Table 6.** Association between TyG index and postoperative stroke in sensitivity analyses.

| **Population** | | **Unadjusted** | | **Model 3**^a^ | |
| --- | --- | --- | --- | --- | --- |
|  |  | OR (95% CI) | *P-value* | OR (95% CI) | *P-value* |
| Condition 1 | Non-imputed original dataset | 2.008 (1.311-3.073) | 0.001 | 2.025 (1.197-3.429) | 0.009 |
| Condition 2 | Simple imputation original dataset | 2.008 (1.311-3.073) | 0.001 | 2.005 (1.218-3.301) | 0.006 |
| Condition 3 | Excluding participants out of 5 years | 2.002 (1.302-3.077) | 0.001 | 1.955 (1.179-3.242) | 0.009 |
| Condition 4 | Excluding participants with history of smoking | 2.672 (1.554-4.594) | 0.001 | 2.427 (1.260-4.673) | 0.008 |
| Condition 5 | Excluding participants with history of drinking | 2.350 (1.372-4.026) | 0.002 | 2.137 (1.152-3.964) | 0.016 |
| Condition 6 | Excluding participants diagnosed with preoperative HE | 2.045 (1.198-3.491) | 0.008 | 2.581 (1.352-4.927) | 0.004 |
| Condition 7 | Excluding participants not receiving piggyback liver transplants | 1.985 (1.301-3.028) | 0.001 | 1.924 (1.167-3.171) | 0.011 |

**Abbreviation:** OR, odds ratio; CI, confidence intervals; HE, hepatic encephalopathy.

^a^Model 3 was adjusted for age, sex, BMI, ASA classification, hypertension, diabetes, renal insufficiency, HE, MELD score, hemodialysis, HB, WBC, platelet, day-or-night surgery, surgery duration, massive transfusion, massive blood losing, uriry oliguria and intraoperative cardiac arrest.
