# Supplementary material for: The triglyceride-glucose index: a novel predictor of stroke and all-cause mortality in liver transplantation recipients
Source: Cardiovasc Diabetol. 2024 Jan 13;23:27. doi: 10.1186/s12933-023-02113-x (PMC10787491; doi:10.1186/s12933-023-02113-x)
Supplement: Supplementary file 7 — Supplementary Material 7: Supplementary Table 7. Postoperative relative outcomes of patients categorized by TyG index [file 12933_2023_2113_MOESM7_ESM.docx]

**Supplementary Table 7.** Postoperative relative outcomes of patients categorized by TyG index^a^.

|  | **All (N=780)** | **T1-T2 (N=521)** | **T3 (N= 259)** | ***P****-value* |
| --- | --- | --- | --- | --- |
| Stroke | 42 (5.38%) | 18 (3.45%) | 24 (9.27%) | **<0.001** |
| Hospital morality | 43 (5.54%) | 22 (4.26%) | 21 (8.11%) | **0.027** |
| 1-year morality | 103 (13.21%) | 59 (11.32%) | 44 (16.99%) | **0.028** |
| 3-year morality | 123 (15.77%) | 71 (13.63%) | 52 (20.08%) | **0.020** |
| Secondary operation | 54 (6.92%) | 31 (5.95%) | 23 (8.88%) | 0.129 |
| Hemodialysis | 211 (29.35%) | 109 (22.57%) | 102 (43.22%) | **<0.001** |
| Hospitalization cost (yuan) | 301560.52 (250408.12-390470.54) | 294967.9 (248486.6-371464.2) | 324448.1 (256516.6-417913.4) | **0.001** |
| Postoperative length of stay (d) | 22.00 (17.00-31.00) | 22.00 (17.00-31.00) | 24.00 (18.00-32.00) | 0.180 |
| Postoperative ICU stay (d) | 2.90 (1.80-4.77) | 2.80 (1.80-4.60) | 3.40 (1.70-5.65) | **0.013** |
| **Postoperative complication** |  |  |  |  |
| PPCs | 530 (67.95%) | 355 (68.14%) | 175 (67.57%) | 0.872 |
| PND | 266 (34.10%) | 173 (33.21%) | 93 (35.91%) | 0.453 |
| AKI | 390 (54.24%) | 243 (50.52%) | 147 (61.76%) | **0.004** |
| Sepsis | 217 (27.82%) | 140 (26.87%) | 77 (29.73%) | 0.401 |
| Hepatorenal syndrome | 27 (3.46%) | 10 (1.92%) | 17 (6.56%) | **0.001** |

**Note:** Data were expressed as mean (standard deviation), median (interquartile range) or n (%). Bold data indicates significance at <0.05. TyG index: T1 (< 7.92), T2 (7.92-8.53), T3 (>8.53).

**Abbreviation:** PPCs, postoperative pulmonary complications; PND, perioperative neurocognitive disorders; AKI, acute kidney injury.
