# Supplementary material for: The triglyceride-glucose index: a novel predictor of stroke and all-cause mortality in liver transplantation recipients
Source: Cardiovasc Diabetol. 2024 Jan 13;23:27. doi: 10.1186/s12933-023-02113-x (PMC10787491; doi:10.1186/s12933-023-02113-x)
Supplement: Supplementary file 8 — Supplementary Material 8: Supplementary Table 8. Supplementary Online Content [file 12933_2023_2113_MOESM8_ESM.docx]

**Supplementary Online Content**

**The Triglyceride-Glucose Index: A Novel Predictor of Stroke and All-cause Mortality in Liver Transplantation Recipients**

**Supplementary Table 1** Overview of missing values in the original data.

**Supplementary Table 2** All baseline clinical characteristics of patients stratified by stroke.

**Supplementary Table 3** Binary logistic regression analysis of the factors influencing stroke of the study population.

**Supplementary Table 4** Collinearity diagnostics by variance expansion factor (VIF).

**Supplementary Table 5** The definitions of confounders.

**Supplementary Table 6** Association between TyG index and postoperative stroke in sensitivity analyses.

**Supplementary Table 7** Postoperative relative outcomes of patients categorized by TyG index.

**Supplementary Table 1.** Overview of missing values in the original data.

| **Variables** | **Missing number (%)** | **Variables** | **Missing number (%)** |
| --- | --- | --- | --- |
| Age | 0 | HE | 0 |
| Sex | 0 | Metabolic acidosis | 0 |
| Height | 1.7 | **Treatments** |  |
| Weight | 1.7 | Mechanical ventilation | 0 |
| BMI | 3.3 | Hemodialysis | 0 |
| ASA | 0 | PE | 0 |
| Smoking | 0 | **Laboratory tests** |  |
| Alcoholism | 0 | TYG index | 0 |
| Drug abuse | 0 | Hemoglobin | 7.3 |
| Previous surgery | 0 | WBC | 1.0 |
| Child Pugh score | 7.9 | Platelet | 1.2 |
| SOFA | 8.5 | Amylase | 6.5 |
| MELD | 5.8 | TG | 0 |
| Comorbidities | 0 | FBG | 0 |
| hepatitis B | 0 | TC | 0 |
| hepatitis C | 0 | HDL | 0 |
| Liver cancer | 0 | LDL | 0 |
| Alcoholic liver disease | 0 | PT | 7.4 |
| Cirrhosis | 0 | INR | 1.7 |
| Portal hypertension | 0 | FIB | 1.2 |
| Hypersplenism | 0 | ALT | 8.6 |
| Fever | 0 | AST | 8.7 |
| Renal insufficiency | 0 | TBIL | 7.2 |
| Diabetes | 0 | IBIL | 7.1 |
| Hypertension | 0 | SCr | 0 |
| PAH | 0 | BUN | 7.6 |
| Respiratory disease | 0 | Albumin | 4.1 |
| Ammonia | 5.4 | **Outcomes** |  |
| **Intraoperative indicators** |  | Secondary operation | 0 |
| Emergency surgery | 0 | Hemodialysis | 0 |
| Day-or-Night surgery | 0 | Hospitalization cost (yuan) | 5.4 |
| Surgery duration | 0 | Total length of stay (d) | 0 |
| Blood group incompatibility | 0 | Postoperative length of stay | 0 |
| Donor type | 0 | Postoperative ICU stay (d) | 0 |
| Surgery technique | 0 | PPCs | 0 |
| Anhepatic phase duration | 8.7 | PND | 0 |
| Cold ischemic duration | 8.3 | AKI | 0 |
| Massive transfusion | 0 | Sepsis | 0 |
| Massive blood losing | 0 | Hepatorenal syndrome | 0 |
| Uriry oliguria | 0 |  |  |
| Electrolyte imbalance | 0 |  |  |
| Cardiac arrest | 0 |  |  |
| Hyperlactatemia | 0 |  |  |
| Vasopressor administration | 0 |  |  |

**Abbreviation:** BMI, body mass index; ASA, American Society of Anesthesiologists; SOFA, sequential organ failure assessment score; MELD, model for end-stage liver disease score; HE, hepatic encephalopathy; PE, plasma exchange; TyG, triglyceride-glucose index; WBC, white blood cell; TG, triglyceride; FBG, fasting blood glucose;; TC, total cholesterol; HDL, high density lipoprotein; LDL, low density lipoprotein; PT, prothrombin time; INR, international normalized ratio; FIB, fibrinogen; ALT, alanine aminotransferase; AST, aspartate aminotransferase; TBIL, total bilirubin; IBIL, indirect bilirubin; SCr, serum creatinine; BUN, blood urea nitrogen; PPCs, postoperative pulmonary complications; PND, perioperative neurocognitive dysfunction; AKI, acute kidney insufficiency.

**Supplementary Table 2.** All baseline clinical characteristics of patients stratified by stroke.

|  | **All (N=780)** | **Non-Stroke (N=738)** | **Stroke (N=42)** | ***P****-value* |
| --- | --- | --- | --- | --- |
| Age (years) | 49.00 (42.00-56.00) | 49.00 (41.25-55.75) | 52.00 (42.50-61.00) | 0.094 |
| Sex (male) | 680 (87.18%) | 643 (87.1%) | 37 (88.1%) | 0.855 |
| Height (cm) | 168.00 (165.00-172.00) | 168.00 (165.00-172.00) | 168.00 (165.25-172.00) | 0.894 |
| Weight (kg) | 65.00 (60.00-69.00) | 65.00 (60.00-69.00) | 65.00 (60.00-65.00) | 0.732 |
| BMI | 22.70 (21.00-24.42) | 22.70 (20.92-24.37) | 22.50 (21.22-24.78) | 0.856 |
| ASA |  |  |  | **<0.001** |
| 2 | 71 (9.10%) | 70 (9.5%) | 1 (2.4%) |  |
| 3 | 706 (90.51%) | 668 (90.5%) | 38 (90.5%) |  |
| 4 | 3 (0.38%) | 0 (0.0%) | 3 (7.1%) |  |
| Smoking | 243 (31.15%) | 225 (30.5%) | 18 (42.9%) | 0.092 |
| Alcoholism | 200 (25.64%) | 184 (24.9%) | 16 (38.1%) | 0.057 |
| Drug abuse | 4 (0.51%) | 4 (0.5%) | 0 (0.0%) | 0.632 |
| Previous surgery | 53 (6.79%) | 49 (6.6%) | 4 (9.5%) | 0.47 |
| Child Pugh score | 10.00 (8.00-11.00) | 10.00 (8.00-11.00) | 10.00 (9.00-11.00) | **0.035** |
| SOFA | 11.00 (9.00-13.00) | 11.00 (9.00-13.00) | 12.00 (10.25-14.00) | **0.042** |
| MELD | 23.00 (22.00-34.00) | 22.00 (22.00-34.00) | 31.50 (23.25-39.50) | **0.001** |
| Comorbidities |  |  |  |  |
| hepatitis B | 594 (76.15%) | 567 (76.8%) | 27 (64.3%) | 0.064 |
| hepatitis C | 18 (2.31%) | 18 (2.4%) | 0 (0.0%) | 0.306 |
| Liver cancer | 317 (40.64%) | 306 (41.5%) | 11 (26.2%) | 0.05 |
| Alcoholic liver disease | 55 (7.05%) | 48 (6.5%) | 7 (16.7%) | **0.012** |
| Cirrhosis | 642 (82.31%) | 617 (83.6%) | 25 (59.5%) | **<0.001** |
| Portal hypertension | 426 (54.62%) | 411 (55.7%) | 15 (35.7%) | **0.011** |
| Hypersplenism | 416 (53.33%) | 404 (54.7%) | 12 (28.6%) | **<0.001** |
| Fever | 95 (12.18%) | 85 (11.5%) | 10 (23.8%) | **0.018** |
| Renal insufficiency | 207 (26.54%) | 191 (25.9%) | 16 (38.1%) | **0.081** |
| Diabetes | 109 (13.97%) | 98 (13.3%) | 11 (26.2%) | **0.019** |
| Hypertension | 67 (8.59%) | 63 (8.5%) | 4 (9.5%) | 0.824 |
| Pulmonary arterial hypertension | 3 (0.38%) | 3 (0.4%) | 0 (0.0%) | 0.679 |
| Respiratory disease | 207 (26.54%) | 191 (25.9%) | 16 (38.1%) | 0.081 |
| HE | 156 (20.00%) | 140 (19.0%) | 16 (38.1%) | **0.003** |
| Metabolic acidosis | 313 (40.13%) | 292 (39.6%) | 21 (50.0%) | 0.18 |
| **Treatments** |  |  |  |  |
| Mechanical ventilation | 60 (7.69%) | 53 (7.2%) | 7 (16.7%) | **0.025** |
| Hemodialysis | 230 (29.49%) | 205 (27.8%) | 25 (59.5%) | **<0.001** |
| PE | 170 (21.79%) | 151 (20.5%) | 19 (45.2%) | **<0.001** |
| **Laboratory tests** |  |  |  |  |
| TYG index | 8.23 (7.78-8.72) | 8.21 (7.76-8.69) | 8.68 (8.14-9.02) | **<0.001** |
| Hemoglobin (g/L) | 101.00 (83.00-122.00) | 102.00 (83.00-122.57) | 90.14 (75.25-106.25) | **0.003** |
| WBC (10^9^/L) | 5.39 (3.58-8.76) | 5.29 (3.55-8.55) | 8.18 (3.96-11.82) | **0.015** |
| Platelet (10^9^/L) | 72.52 (46.00-122.00) | 73.00 (47.00-123.75) | 58.50 (40.00-99.00) | 0.063 |
| Amylase (U/L) | 76.00 (54.00-107.00) | 76.00 (55.00-106.00) | 68.00 (49.00-112.50) | 0.482 |
| TG (mmol/L) | 0.74 (0.55-1.05) | 0.74 (0.55-1.05) | 0.77 (0.60-1.04) | 0.677 |
| FBG (mmol/L) | 5.00 (4.22-6.43) | 4.96 (4.20-6.27) | 6.81 (4.75-10.04) | **<0.001** |
| TC (mmol/L) | 3.03 (2.08-3.93) | 3.06 (2.10-4.00) | 2.46 (1.90-3.41) | **0.01** |
| HDL (mmol/L) | 0.47 (0.15-0.91) | 0.48 (0.15-0.93) | 0.34 (0.13-0.60) | **0.02** |
| LDL (mmol/L) | 1.50 (0.96-2.32) | 1.53 (0.96-2.33) | 1.25 (0.94-1.76) | 0.077 |
| PT (s) | 17.75 (14.30-26.40) | 17.60 (14.30-26.40) | 20.15 (15.80-25.80) | 0.068 |
| INR | 1.73 (1.22-2.83) | 1.71 (1.21-2.80) | 2.29 (1.56-3.80) | 0.338 |
| FIB (g/L) | 1.59 (1.05-2.65) | 1.62 (1.07-2.67) | 1.27 (0.82-1.89) | **0.004** |
| ALT (U/L) | 54.90 (27.00-116.00) | 54.00 (27.00-116.00) | 83.85 (29.25-119.75) | **0.005** |
| AST (U/L) | 85.00 (43.00-150.00) | 82.00 (43.00-149.09) | 110.00 (54.25-158.90) | 0.236 |
| TBIL (μmol/L) | 126.55 (24.64-434.47) | 120.14 (24.41-433.00) | 313.52 (67.90-516.54) | 0.137 |
| IBIL (μmol/L) | 38.83 (10.80-137.99) | 36.40 (10.50-132.62) | 111.60 (25.02-188.40) | **0.026** |
| SCr (μmol/L) | 73.00 (60.00-92.00) | 73.00 (60.00-91.00) | 73.50 (59.50-148.75) | **0.013** |
| BUN (mmol/L) | 4.79 (3.51-6.92) | 4.79 (3.51-6.96) | 4.76 (3.40-6.74) | 0.27 |
| Albumin (g/L) | 35.58 (4.91) | 35.63 (4.96) | 34.83 (3.92) | 0.305 |
| Ammonia (μmol/L) | 26.09 (18.61-36.77) | 26.01 (18.58-36.15) | 28.17 (19.65-48.13) | 0.192 |
| **Intraoperative indicators** | |  |  |  |
| Emergency surgery | 161 (20.64%) | 155 (21.0%) | 6 (14.3%) | 0.295 |
| Day-or-Night surgery | 276 (35.38%) | 251 (34.0%) | 25 (59.5%) | **<0.001** |
| Surgery duration | 521.00 (470.00-580.00) | 520.00 (470.00-580.00) | 532.50 (486.00-622.25) | 0.174 |
| Blood group incompatibility | 104 (13.33%) | 95 (12.9%) | 9 (21.4%) | 0.115 |
| Donor type |  |  |  | **0.005** |
| DBD | 471 (60.38%) | 453 (61.4%) | 18 (42.9%) |  |
| DCD | 301 (38.59%) | 279 (37.8%) | 22 (52.4%) |  |
| DBCD | 8 (1.03%) | 6 (0.8%) | 2 (4.8%) |  |
| Surgery technique |  |  |  | 0.147 |
| Piggyback | 718 (92.05%) | 676 (91.6%) | 42 (100.0%) |  |
| Split liver | 33 (4.23%) | 33 (4.5%) | 0 (0.0%) |  |
| Standard | 29 (3.72%) | 29 (3.9%) | 0 (0.0%) |  |
| Anhepatic phase duration | 46.00 (40.00-54.00) | 46.00 (40.00-54.00) | 46.50 (38.50-56.75) | 0.738 |
| Cold ischemic duration | 360.00 (330.00-410.00) | 360.00 (330.00-410.00) | 360.00 (330.00-420.00) | 0.759 |
| Massive transfusion | 231 (29.62%) | 210 (28.5%) | 21 (50.0%) | **0.003** |
| Massive blood losing | 37 (4.74%) | 30 (4.1%) | 7 (16.7%) | **<0.001** |
| Urinary oliguria | 32 (4.10%) | 25 (3.4%) | 7 (16.7%) | **<0.001** |
| Electrolyte imbalance | 302 (38.72%) | 286 (38.8%) | 16 (38.1%) | 0.932 |
| Cardiac arrest | 16 (2.036%) | 13 (1.762%) | 3 (7.143%) | **0.017** |
| Hyperlactatemia | 405 (51.92%) | 388 (52.6%) | 17 (40.5%) | 0.127 |
| Vasopressor administration | 760 (97.44%) | 719 (97.4%) | 41 (97.6%) | 0.938 |

**Note:** Data were expressed as mean (standard deviation), median (interquartile range) or n (%). Bold data indicates significance at <0.05.

**Abbreviation:** BMI, body mass index; ASA, American Society of Anesthesiologists; SOFA, sequential organ failure assessment score; MELD, model for end-stage liver disease score; HE, hepatic encephalopathy; PE, plasma exchange; TyG, triglyceride-glucose index; WBC, white blood cell; TG, triglyceride; FBG, fasting blood glucose;; TC, total cholesterol; HDL, high density lipoprotein; LDL, low density lipoprotein; PT, prothrombin time; INR, international normalized ratio; FIB, fibrinogen; ALT, alanine aminotransferase; AST, aspartate aminotransferase; TBIL, total bilirubin; IBIL, indirect bilirubin; SCr, serum creatinine; BUN, blood urea nitrogen; DBD, donation after brain death; DCD, donation after circulatory death; DBCD, donation after brain death followed by circulatory death.

**Supplementary Table 3.** Binary logistic regression analysis of the factors influencing stroke of the study population.

| **Variables** | **OR/β** | | **Lower 95% CI** | **Upper 95% CI** | **P-value** |
| --- | --- | --- | --- | --- | --- |
| Age | 1.030 | | 0.999 | 1.061 | 0.055 |
| Sex | 1.093 | | 0.419 | 2.850 | 0.855 |
| BMI | 0.972 | | 0.878 | 1.076 | 0.588 |
| Smoking | 1.710 | | 0.910 | 3.214 | 0.0956 |
| Alcoholism | 1.853 | | 0.972 | 3.531 | 0.061 |
| Previous surgery | 1.481 | | 0.508 | 4.316 | 0.473 |
| Child Pugh score | 1.204 | | 1.025 | 1.415 | 0.023 |
| SOFA | 1.132 | | 1.011 | 1.266 | 0.031 |
| MELD | 1.061 | | 1.022 | 1.101 | **0.002** |
| Alcoholic liver disease | 2.875 | | 1.213 | 6.812 | 0.016 |
| Cirrhosis | 0.288 | | 0.151 | 0.551 | 0.021 |
| Portal hypertension | 0.442 | | 0.231 | 0.844 | 0.013 |
| Hypersplenism | 0.331 | | 0.166 | 0.656 | **0.002** |
| Fever | 2.401 | | 1.139 | 5.058 | 0.021 |
| Renal insufficiency | 1.762 | | 0.925 | 3.356 | 0.084 |
| Diabetes | 2.317 | | 1.128 | 4.761 | 0.022 |
| Hypertension | 1.128 | | 0.389 | 3.262 | 0.824 |
| HE | 2.629 | | 1.373 | 5.032 | **0.004** |
| Mechanical ventilation | 2.585 | | 1.096 | 6.098 | 0.030 |
| Hemodialysis | 3.824 | | 2.023 | 7.228 | **<0.001** |
| PE | 3.211 | | 1.705 | 6.050 | **0.003** |
| TYG index | 2.008 | | 1.311 | 3.073 | **0.001** |
| Hemoglobin | 0.979 | | 0.965 | 0.992 | **0.002** |
| WBC | 1.079 | | 1.029 | 1.131 | **0.002** |
| Platelet | 0.995 | | 0.989 | 1.001 | 0.068 |
| TC | 0.741 | | 0.574 | 0.958 | 0.022 |
| HDL | 0.431 | | 0.192 | 0.970 | 0.041 |
| FIB | 0.649 | | 0.457 | 0.922 | 0.015 |
| ALT | 1.000 | | 0.999 | 1.002 | 0.558 |
| IBIL | 1.003 | | 1.000 | 1.006 | 0.042 |
| SCr | 1.003 | | 1.000 | 1.006 | 0.026 |
| Day-or-Night surgery | | |  |  |  |
| Day | Ref. | | Ref. | Ref. | Ref. |
| Night | 2.853 | | 1.513 | 5.383 | **0.001** |
| Surgery duration | 1.001 | | 0.999 | 1.003 | 0.359 |
| Donor type |  | |  |  |  |
| DBD | Ref. | | Ref. | Ref. | Ref. |
| DCD | 1.984 | | 1.046 | 3.766 | 0.036 |
| DBCD | 8.389 | | 1.582 | 44.484 | 0.012 |
| Massive transfusion | 2.514 | | 1.345 | 4.700 | **0.004** |
| Massive blood losing | 4.720 | | 1.938 | 11.493 | **<0.001** |
| Urinary oliguria | | 5.704 | 2.309 | 14.089 | **<0.001** |
| Cardiac arrest | 4.290 | | 1.174 | 15.680 | 0.027 |

**Abbreviation:** BMI, body mass index; SOFA, sequential organ failure assessment score; MELD, model for end-stage liver disease score; HE, hepatic encephalopathy; PE, plasma exchange; TyG, triglyceride-glucose index; WBC, white blood cell; FBG, fasting blood glucose; HDL, high density lipoprotein; FIB, fibrinogen; ALT, alanine aminotransferase; IBIL, indirect bilirubin; SCr, serum creatinine; DBD, donation after brain death; DCD, donation after circulatory death; DBCD, donation after brain death followed by circulatory death.

**Supplementary Table 4.** Collinearity diagnostics by variance expansion factor (VIF).

| variables | VIF |
| --- | --- |
| Age | 1.2 |
| Sex | 1.1 |
| BMI | 1.1 |
| ASA | 1.1 |
| MELD | 1.8 |
| Diabetes | 1.3 |
| Hypertension | 1.3 |
| Renal insufficiency | 1.3 |
| HE | 1.4 |
| Hemodialysis | 1.5 |
| TYG index | 1.9 |
| Hemoglobin | 1.4 |
| WBC | 1.4 |
| Platelet | 1.3 |
| Day-or-Night surgery | 1 |
| Surgery duration | 1.1 |
| Massive transfusion | 1.4 |
| Massive blood losing | 1.2 |
| Urinary oliguria | 1.1 |
| Cardiac arrest | 1.6 |

**Note:** VIF for a variable = 1/(1-R2), where R2 is the R-squared of the regression model of that variable against all other variables (e.g. X1=X2+X3+...). All covariates with VIF ≤ 5.

**Abbreviation:** BMI, body mass index; ASA, American Society of Anesthesiologists; MELD, model for end-stage liver disease score; HE, hepatic encephalopathy; TyG, triglyceride-glucose index; WBC, white blood cell.

**Supplementary Table 5.** The definitions of confounders.

| **Confounder** | **Definition** |
| --- | --- |
| **Patient-related confounders** | |
| Age | age in the year of surgery |
| Sex | male or female |
| BMI (kg m^-2^) | calculated by the body weight and height measured at admission |
| ASA classification | assessed by the anaesthesiologists in charge of the case |
| Hypertension | with a previous diagnosis of hypertension, including well-controlled hypertension |
| Diabetes | with a previous diagnosis of diabetes mellitus, including well-controlled diabetes |
| HE | with a previous diagnosis of hepatic encephalopathy, including covert hepatic encephalopathy |
| MELD score | calculated by the SCr, TBIL and INR before surgery |
| Hemodialysis | Renal insufficiency combined with fluid and electrolyte imbalance requiring any form of hemodialysis treatment including continuous renal replacement therapy |
| Hemoglobin | / |
| WBC | / |
| Platelet | / |
| Renal insufficiency | The ratio of preoperative maximum SCr to minimum SCr was over 1.5 in the last test before surgery |
| **Surgery-related confounders** | |
| Day-or-Night surgery | whether the timing of the surgery was during the day (8:00 AM- 8:00 PM) or at night (8:00 PM- 8:00 AM) |
| Surgery duration | duration of the liver transplantation |
| Massive transfusion | transfusion of more than 20 units of red blood cells or transfusion of blood components exceeding 1 to 1.5 times the patient's own blood volume in 24 hours. |
| Massive blood losing | loss of a circulating blood volume in 24 hours, or half the circulating blood volume within 3 hours, or a bleeding rate greater than 150 mL/min. |
| Uriry oliguria | intraoperative urine output ≤0.5ml/kg/h |
| Intra-liver transplantation cardiac arrest | any cardiac arrest that occurs during surgery |

**Abbreviation:** BMI, body mass index; ASA, American Society of Anesthesiologists; HE, hepatic encephalopathy; MELD, model for end-stage liver disease score; WBC, white blood cell.

**Supplementary Table 6.** Association between TyG index and postoperative stroke in sensitivity analyses.

| **Population** | | **Unadjusted** | | **Model 3**^a^ | |
| --- | --- | --- | --- | --- | --- |
|  |  | OR (95% CI) | *P-value* | OR (95% CI) | *P-value* |
| Condition 1 | Non-imputed original dataset | 2.008 (1.311-3.073) | 0.001 | 2.025 (1.197-3.429) | 0.009 |
| Condition 2 | Simple imputation original dataset | 2.008 (1.311-3.073) | 0.001 | 2.005 (1.218-3.301) | 0.006 |
| Condition 3 | Excluding participants out of 5 years | 2.002 (1.302-3.077) | 0.001 | 1.955 (1.179-3.242) | 0.009 |
| Condition 4 | Excluding participants with history of smoking | 2.672 (1.554-4.594) | 0.001 | 2.427 (1.260-4.673) | 0.008 |
| Condition 5 | Excluding participants with history of drinking | 2.350 (1.372-4.026) | 0.002 | 2.137 (1.152-3.964) | 0.016 |
| Condition 6 | Excluding participants diagnosed with preoperative HE | 2.045 (1.198-3.491) | 0.008 | 2.581 (1.352-4.927) | 0.004 |
| Condition 7 | Excluding participants not receiving piggyback liver transplants | 1.985 (1.301-3.028) | 0.001 | 1.924 (1.167-3.171) | 0.011 |

**Abbreviation:** OR, odds ratio; CI, confidence intervals; HE, hepatic encephalopathy.

^a^Model 3 was adjusted for age, sex, BMI, ASA classification, hypertension, diabetes, renal insufficiency, HE, MELD score, hemodialysis, HB, WBC, platelet, day-or-night surgery, surgery duration, massive transfusion, massive blood losing, uriry oliguria and intraoperative cardiac arrest.

**Supplementary Table 7.** Postoperative relative outcomes of patients categorized by TyG index^a^.

|  | **All (N=780)** | **T1-T2 (N=521)** | **T3 (N= 259)** | ***P****-value* |
| --- | --- | --- | --- | --- |
| Stroke | 42 (5.38%) | 18 (3.45%) | 24 (9.27%) | **<0.001** |
| Hospital morality | 43 (5.54%) | 22 (4.26%) | 21 (8.11%) | **0.027** |
| 1-year morality | 103 (13.21%) | 59 (11.32%) | 44 (16.99%) | **0.028** |
| 3-year morality | 123 (15.77%) | 71 (13.63%) | 52 (20.08%) | **0.020** |
| Secondary operation | 54 (6.92%) | 31 (5.95%) | 23 (8.88%) | 0.129 |
| Hemodialysis | 211 (29.35%) | 109 (22.57%) | 102 (43.22%) | **<0.001** |
| Hospitalization cost (yuan) | 301560.52 (250408.12-390470.54) | 294967.9 (248486.6-371464.2) | 324448.1 (256516.6-417913.4) | **0.001** |
| Postoperative length of stay (d) | 22.00 (17.00-31.00) | 22.00 (17.00-31.00) | 24.00 (18.00-32.00) | 0.180 |
| Postoperative ICU stay (d) | 2.90 (1.80-4.77) | 2.80 (1.80-4.60) | 3.40 (1.70-5.65) | **0.013** |
| **Postoperative complication** |  |  |  |  |
| PPCs | 530 (67.95%) | 355 (68.14%) | 175 (67.57%) | 0.872 |
| PND | 266 (34.10%) | 173 (33.21%) | 93 (35.91%) | 0.453 |
| AKI | 390 (54.24%) | 243 (50.52%) | 147 (61.76%) | **0.004** |
| Sepsis | 217 (27.82%) | 140 (26.87%) | 77 (29.73%) | 0.401 |
| Hepatorenal syndrome | 27 (3.46%) | 10 (1.92%) | 17 (6.56%) | **0.001** |

**Note:** Data were expressed as mean (standard deviation), median (interquartile range) or n (%). Bold data indicates significance at <0.05. TyG index: T1 (< 7.92), T2 (7.92-8.53), T3 (>8.53).

**Abbreviation:** PPCs, postoperative pulmonary complications; PND, perioperative neurocognitive disorders; AKI, acute kidney injury.
